# From Lab to Field: Context-Dependent Impacts of *Pseudomonas*-Produced 2,4-Diacetylphloroglucinol on Soil Microbial Ecology

**DOI:** 10.3390/biom15111578

**Published:** 2025-11-10

**Authors:** Anastasia V. Teslya, Artyom A. Stepanov, Darya V. Poshvina, Ivan S. Petrushin, Alexey S. Vasilchenko

**Affiliations:** 1 Laboratory of Antimicrobial Resistance, Institute of Environmental and Agricultural Biology (X-BIO), Tyumen State University, Tyumen 625003, Russia; 2 Laboratory of Biochemistry and Ecology of Microorganisms, All-Russian Institute of Plant Protection, Pushkin 196608, Russia; 3 Irkutsk State University, Irkutsk 664033, Russia; ivan.kiel@gmail.com; 4 Siberian Institute of Plant Physiology and Biochemistry, Siberian Branch of the Russian Academy of Sciences, Irkutsk 664033, Russia

**Keywords:** soil health, soil microbiome, plant growth-promoting rhizobacteria, *Pseudomonas fluorescence*, antibiotic, soil enzymes, plant protection, biosecurity

## Abstract

The secondary metabolite 2,4-diacetylphloroglucinol (2,4-DAPG), which is produced by *Pseudomonas* bacteria, is a potent antimicrobial agent with well-documented properties that suppress phytopathogens. However, its broader ecological impact on soil microbial communities is not understood. Through a combination of controlled microcosm and field trials, we have demonstrated that the effects of 2,4-DAPG are highly context-dependent. Laboratory exposure (10 mg kg^−1^) altered the abundance of 8.53% of bacterial and 6.91% of fungal amplicon sequence variants, and simplified the bacterial co-occurrence networks (reduced number of nodes and links). In contrast, field conditions amplified bacterial sensitivity (the Shannon index decreased from 4.77 to 4.17, *p* < 0.05) but maintained fungal stability (Shannon index varied from 3.93 to 3.97, *p* > 0.05); these conditions affected a smaller proportion of fungal ASVs (4.23%). Taxonomic analysis revealed consistent suppression of fungi of the Mucoromycota (e.g., *Mortierella*) and context-dependent shifts in bacteria, with an enrichment of Bacillota (e.g., *Bacillus*, *Paenibacillus*) in the laboratory but not in the field. Enzymatic responses revealed a dose-dependent activation of the C-cycle, with up to 7.4-fold increases in the laboratory and up to a 10.5-fold increase in the field. P- and N- cycles showed more complex dynamics, with acid phosphatase activity increasing 3.8-fold in laboratory conditions and recovering from initial suppression to an increase of 144% in field conditions, while N-acetylglucosaminidase activity increased and L-leucine aminopeptidase decreased under laboratory conditions. Our results suggest that the response of microorganisms to 2,4-DAPG in natural soils is reduced, probably due to functional redundancy and pre-adaptation to abiotic stresses. This difference between laboratory and field studies warns against extrapolating data from controlled experiments to predict outcomes in agricultural ecosystems, and emphasizes the need for a context-specific evaluation of biocontrol agents.

## 1. Introduction

2,4-Diacetylphloroglucinol (2,4-DAPG) is a key secondary metabolite produced by plant-protective Pseudomonas bacteria, giving them a competitive advantage in the rhizosphere. 2,4-DAPG exhibits a wide range of antimicrobial activities [1], and the genes for its biosynthetic operon (*phlACBD*) are not only widely distributed among pseudomonads, but also beyond this genus [2]. Since its discovery over 50 years ago [3], 2,4-DAPG has been recognized for its crucial role in suppressing various phytopathogens, including fungi (e.g., *Phytophthora infestans*, *Fusarium oxysporum*, *Zymoseptoria tritici* [4,5,6], bacteria (e.g., *Ralstonia solanacearum*, *Xanthomonas* spp. [7,8,9], and nematodes [10]. The critical role of 2,4-DAPG in biocontrol is confirmed by the loss of protective activity in bacterial strains with knocked-out *phl* genes [11]. While this broad-spectrum activity is key to its efficacy, it simultaneously poses a potential threat to non-target microorganisms. Consequently, the impact of 2,4-DAPG on soil microbiomes, particularly unculturable yet agronomically important taxa, remains poorly understood and associated with risk.

The biosynthesis of 2,4-DAPG in *Pseudomonas* spp. is governed by the conserved *phlACBD* operon, which encodes a type III polyketide synthase system that sequentially converts malonyl-CoA into the phloroglucinol backbone [12]. This secondary metabolite not only functions as a potent antimicrobial but also serves as a signaling molecule that regulates its own production and influences microbial community interactions [13]. As a polyphenolic compound, the fate and persistence of 2,4-DAPG in soil are likely governed by common processes for its chemical class. In soil, phenolics can exist in dissolved, sorbed, or polymerized forms, and their bioavailability is regulated by sorption to soil particles through hydrophobic and hydrogen bonding [14,15]. Their degradation is primarily driven by specialized microorganisms, such as fungi (Basidiomycetes and Ascomycetes), and bacteria (*Pseudomonas*), which use extracellular enzymes and metabolic pathways such as the β-ketoadipate pathway and dioxygenase-initiated ring cleavage [16]. Therefore, the actual ecological impact of 2,4-DAPG is inherently context-dependent, shaped not only by its inherent toxicity but also by local soil conditions that govern its bioavailability and persistence.

The potential for non-targeted effects is a major concern in the use of broad-spectrum antimicrobials. For instance, the use of broad-spectrum synthetic fungicides (e.g., triazoles, strobilurins) frequently results in a reduction in the taxonomic diversity and functional activity of soil microbiomes [17,18,19]. In contrast, certain biological control agents can positively influence the structure and function of microbial community [20]. The ecological impact of 2,4-DAPG, however, places it somewhere on this spectrum and is a critical open question.

While successful biological control is a complex process involving various factors, such as siderophores and other antimicrobial compounds, as well as the induction of plant defense mechanisms, our study specifically focuses on the ecological role of 2,4-DAPG. By adopting a reductionist approach, we can isolate and determine the specific contribution of this key metabolite to the overall impact of biocontrol *Pseudomonas* on soil microbial communities.

Therefore, to provide a holistic understanding of the ecological role of 2,4-DAPG, we conducted an integrated laboratory and field study that goes beyond its established pathogen suppression effects. We quantified its impact on the non-target soil microbiome by measuring functional shifts (basal respiration, microbial biomass, and hydrolytic enzyme activity) and structural changes in bacterial and fungal communities. We tested two central hypotheses: first, that 2,4-DAPG exerts a selective pressure on a narrow subset (~10–15%) of the microbial population, and second, that the response of the community in a natural agroecosystem is fundamentally different from that in a controlled laboratory environment due to the buffering capacity of a complex pre-adapted soil community.

## 2. Materials and Methods

### 2.1. Soil Sample Preparation and Experimental Design

Topsoil (0–10 cm depth) was collected from an agricultural field under gray soil (Luvic Phaeozems according to WRB classification) in Tyumen, Russia (57.0914° N, 65.3740° E). Sampling was performed according to the “envelope” principle (four corners and the center of a defined area. The soil samples were collected, transported to the laboratory within two hours, and immediately processed. Thus, one pooled sample was taken from an agroecosystem (field) and subsamples were prepared for both laboratory and field experiments.

The soil had a loamy texture with the following granulometric composition: 28.5% clay, 27.7% silt, and 43.8% sand. Key chemical properties included: soil organic carbon (SOC) content of 17.4 ± 0.54 g kg^−1^, pH(H_2_O) 6.2, and pH(KCl) 5.6. Visible plant debris and stones were removed, and the soil was sieved (<2 mm) and homogenized prior to the experiment.

The experiment included three treatments, which were set up in both laboratory and field conditions:

Control: Soil amended with a 0.01% (*v*/*v*) aqueous solution of dimethyl sulfoxide (DMSO).
D-1: Soil amended with 2,4-DAPG at a concentration of 1 mg kg^−1^ soil.
D-10: Soil amended with 2,4-DAPG at a concentration of 10 mg kg^−1^ soil.

A high-concentration stock solution of chemically synthesized 2,4-DAPG (purity ≥ 99%) was prepared in dimethyl sulfoxide (DMSO). This stock was then diluted with sterile distilled water to produce final working solutions corresponding to the target soil concentrations of 1 or 10 mg kg^−1^. The final concentration of DMSO in all treatments, including the control, was adjusted to 0.01% (*v*/*v*). The appropriate volume of the respective working solution (or the 0.01% DMSO solution for the control) was sprayed onto the soil while mixing thoroughly in a large container to achieve homogeneous distribution.

The selection of 2,4-DAPG concentrations was designed to represent a gradient of environmentally plausible scenarios. The lower concentration (1 mg kg^−1^) falls within the range of In Situ levels typically produced by pseudomonads in the rhizosphere (0.03–2.77 mg kg^−1^) [21]. This dose allows for the assessment of effects under realistic agronomic conditions. The higher concentration (10 mg kg^−1^) represents a scenario of local antibiotic accumulation in soil microhabitats, such as around a producer colony [22], enabling the evaluation of community resilience and threshold effects under heightened, yet still plausible, antibiotic stress.

The soil moisture content was uniformly adjusted to 60% of its water-holding capacity (WHC) for all treatments. After amendment, the soil was pre-incubated for 24 h at 22 °C to allow for compound equilibration.

For the laboratory experiment, approximately 300 g of prepared soil from each treatment was placed in sterile ziplock bags (one bag = one microcosm). Five independent biological replicates were prepared for each treatment (3 treatments × 5 replicates = 15 microcosms total).

The microcosms were incubated in the dark at 22 °C for 56 days. To maintain stable conditions and minimize water loss, the bags were sealed. Soil moisture was monitored weekly by weight and adjusted to 60% WHC by adding sterile distilled water as needed.

For the field experiment, an equivalent amount of prepared soil (approx. 300 g) from each treatment was placed in perforated containers (food-grade polypropylene, PP5) with multiple 1–2 mm holes to allow for gas exchange and natural water drainage while preventing soil loss. In the field experiment, we adjusted the soil moisture to 60% of WHC and measured it each time after collecting samples for analysis. Five independent biological replicates were prepared for each treatment (3 treatments × 5 replicates = 15 microcosms total).

The containers were buried at a depth of 5 cm within the same agrosystem from which the soil was originally collected (Figure S1). The experiment was conducted from 17 July to 10 September 2023. Meteorological data (precipitation, temperature) recorded during the experimental period are provided in the Figure S2.

The soil samples from each microcosm were collected on days 7, 14, 28, and 56 of incubation. At each sampling event, the collected soil was homogenized and divided into two aliquots: one was immediately frozen at −80 °C for subsequent DNA extraction and molecular analysis, while the other was stored at 4 °C for a maximum of 48 h for analysis of enzymatic activity and physicochemical parameters.

### 2.2. Chemical Analysis

Soil pH was measured potentiometrically in accordance with the international standard ISO 10390:2021 (accessed on 9 November 2025, https://www.iso.org/standard/75243.html). The pH(KCl) was determined in a 1 M KCl suspension, and pH(H_2_O) was measured in a deionized water suspension, both at a soil-to-solution ratio of 1:5 (*w/v*) using an Orion Star A111 pH meter (Thermo Scientific, Waltham, MA, USA). Total carbon (TC) and total nitrogen (TN) content were determined in triplicate by dry combustion using a Vario EL III elemental analyzer (Elementar, Langenselbold, Germany). The TC content was considered equivalent to the soil organic carbon (SOC) content, as carbonates were absent in the studied Luvic Phaeozems.

### 2.3. Determination of Soil Microbiological Properties

Basal (microbial) soil respiration (BR, CO_2_ production) was determined as described in the study [23]. The physiological activity of microorganisms and the intensity of organic matter mineralization in the samples were assessed based on basal soil respiration. Microbial biomass (MB_SIR_) was determined by the substrate-induced respiration (SIR) method [24]. The initial maximum respiration rate of microorganisms (3–5 h) induced by the introduction of an easily oxidizable and universally accessible substrate, glucose, into the soil was measured. The respiratory response is proportional to the total microbial biomass of the soil. CO_2_ production was measured using a gas analyzer LI-830 (LI-COR Biosciences, Lincoln, NE, USA). The BR and SIR results were presented in μg CO_2_ g^−1^ soil h^−1^, and MB_SIR_—in μg C g^−1^ soil. Basal soil respiration and microbial biomass were determined for each biological replicate (3 analytical replicates each).

Based on the collected data, the following ecological and physiological indicators of the microbial community functioning were calculated: the coefficient of basal respiration (QR = BR/SIR); the metabolic coefficient or specific respiration of microbial biomass (*q*CO_2_, =BR/MB_SIR_, μg CO_2_ mg^−1^ MB_SIR_ h^−1^); the share of microbial biomass carbon in organic carbon (MB_SIR_/SOC, %), and the interrelationship between C-use efficiency and the quality of available organic matter in soil (qCO_2_/SOC, μg CO_2_ mg^−1^ MB_SIR_ h^−1^ (g SOC g^−1^ soil^−1^). A detailed description of eco-physiological indicators is presented in the work [25].

### 2.4. Soil Enzyme Activities

The activity of soil hydrolytic enzymes, including those involved in carbon (β-1,4-glucosidase, β-1,4-xylosidase, β-D-1,4-cellobiosidase), nitrogen (β-1,4-N-acetyl-glucosaminidase, L-leucine aminopeptidase), and phosphorus (acid phosphatase) cycling, was quantified using fluorogenically labeled substrates. The detailed methodological approach, including specific substrates and assay conditions, is described in our recent publication [26]. Fluorescence was measured using a Fluorskan Ascent FL microplate reader (Thermo Fisher Scientific, Waltham, MA, USA). All enzyme activities were determined with eight analytical replicates per biological replicate.

### 2.5. Metabarcoding Sample Preparation and Sequencing

Molecular analysis was performed on soil samples collected on day 28 of the experiment. Total genomic DNA was extracted from 250 mg of soil using the Quick-DNA Fecal/Soil Microbe Kit (Zymo Research, Irvine, CA, USA) according to the manufacturer’s protocol.

The DNA concentration and purity were assessed using a Qubit 4.0 Fluorometer with the dsDNA HS Assay Kit (Thermo Fisher Scientific, USA) and a NanoPhotometer N120 (Implen, Munich, Germany), respectively.

For the analysis of the bacterial community, the hypervariable V3-V4 regions of the 16S rRNA gene were amplified using the primer pair 341F (5′-CCTACGGGNGGCWGCAG-3′) and 806R (5′-GGACTACHVGGGTWTCTAAT-3′). For the analysis of the fungal community, the ITS1 region was amplified using the primer pair ITS1F (5′-CTTGGTCATTTAGAGGAAGTAA-3′) and ITS2aR (5′-GCTGCGTTCTTCATCGATGC-3′).

Metabarcoding libraries were prepared using Illumina’s standard protocol “16S Metagenomic Sequencing Library Preparation” (Illumina, San Diego, CA, USA). Briefly, this involved a dual-indexing approach with limited-cycle PCR to attach unique indices and Illumina adapter sequences. The resulting libraries were quantified, normalized, and equimolarly pooled.

The pooled libraries were sequenced on an Illumina MiSeq platform using the MiSeq Reagent Kit v3 (Illumina, San Diego, CA, USA) (600-cycle, 2 × 300 bp paired-end reads) in accordance with the manufacturer’s instructions.

### 2.6. Sequencing Data Processing and Analysis

The processing of the raw sequencing data was performed using a standardized pipeline. The quality of the raw FASTQ files was assessed with Falco v.1.2.1 [26]. Primer and adapter sequences were trimmed using Cutadapt v4.4. Subsequent processing, including demultiplexing, quality filtering, merging of paired-end reads, and removal of chimeric sequences, was conducted using the DADA2 package (v1.28.0) in R, yielding a table of amplicon sequence variants (ASVs) [27].

The taxonomy of the bacterial ASVs was assigned using the Ribosomal Database Project (RDP) classifier against the RDP trainset 18 (release 11.5). Fungal ASVs were classified using the UNITE database (general release 25 July 2023). The resulting ASV tables were used for downstream ecological analyses.

The co-occurrence network was constructed using the MENA pipeline [28] and subsequently visualized and analyzed in Gephi v. 0.10.1.

### 2.7. Statistical Processing

Statistical analyses were performed using Origin 2024 (OriginLab Corporation, Northampton, MA, USA) and Past v5.2.1. The normality of data distribution was assessed using the Shapiro–Wilk test. Based on these results, comparisons between two independent groups were conducted using Student’s *t*-test for normally distributed data or the Mann–Whitney U test for non-normally distributed data. A correction for multiple comparisons was applied using the Sequential Bonferroni (Holm-Bonferroni) method. Alpha diversity indices (Shannon, Chao1) were compared between treatment groups using one-way ANOVA followed by Tukey’s Post Hoc test. A significance level of *p* < 0.05 was applied for all statistical tests.

## 3. Results

### 3.1. Soil Organic Carbon

In a laboratory experiment, the SOC content in untreated soils averaged 17.4 ± 0.54 g kg^−1^ during the entire incubation period (Table 1). On days 7 and 14 of incubation, the SOC content in samples D-1 and D-10 was on average 9.0% higher than that in the control (*p* < 0.001). By day 28, the increase in SOC was 13.8% in samples D-10 (*p* < 0.001). By the end of the incubation period, the carbon content in the soil in the treated samples had decreased, but was still higher than in the control sample (by an average of 6.1%; *p* < 0.05).

In the field experiment, the SOC content in untreated soils remained stable throughout the study period, averaging 15.4 ± 0.71 g kg^−1^ (Table 1). In D-1 microcosms, a decrease in the indicator by 13.6% was noted on day 7 (*p* < 0.01), but by the end of the incubation period, the SOC content was 4.7% higher than the control values (*p* < 0.05). In samples containing D-10, the first two weeks were characterized by an average decrease in SOC of 5.2% (*p* < 0.05), followed by an increase in this indicator. By the end of the experiment, the SOC values were 7.4% higher than in the control group (*p* < 0.05).

### 3.2. Microbiological Activity

#### 3.2.1. Controlled Laboratory Experiments

**Figure 1:**
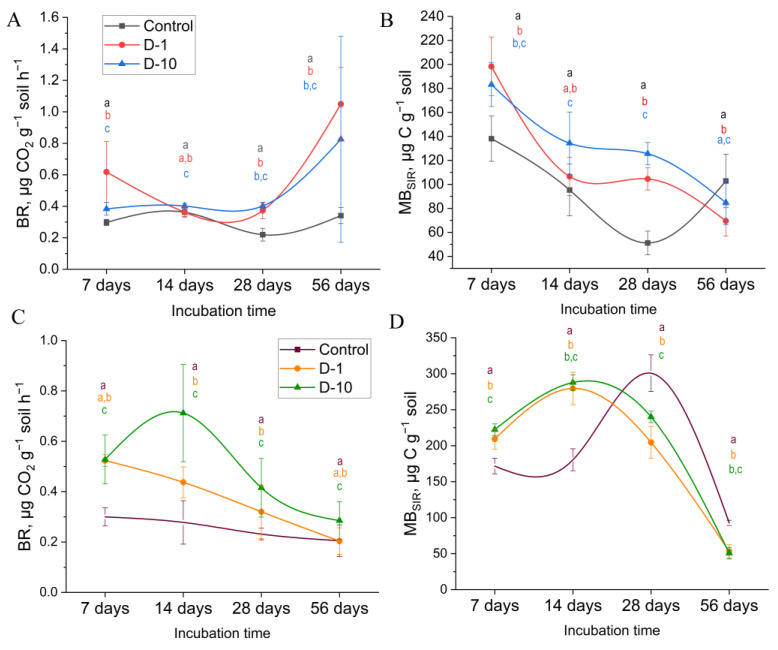
Effects of 2,4-DAPG exposure on soil microbial activity (respiration rate) and microbial biomass content in controlled laboratory microcosms (**A**–**D**) field experiments. Data points represent the mean ± standard deviation from five biological replicates. Different letters indicate statistically significant differences (*p* < 0.05) based on Student’s *t*-test or Mann–Whitney U test (Holm-Bonferroni correction).

Results of the laboratory experiment. On the seventh day, a 107.9% increase in respiratory activity was observed in microbial communities in D-1 microcosms, and a 28.9% increase in D-10 microcosms (*p* < 0.001) (Figure 1A). The increase in microbial biomass in soils treated with 1 and 10 mg kg^−1^ of 2,4-DAPG at was 41.7% and 32.7%, respectively, and was statistically significant (*p* < 0.001).

After two weeks, the BR and MB_SIR_ values were significantly higher in D-10 samples than in the control by 10.9% (*p* < 0.005) and 40.9% (*p* < 0.001), respectively (Figure 1B). By day 14, CO_2_ production by microorganisms in D-1 samples, had decreased by 39.7% (*p* < 0.001), and it corresponded to the control on average (Figure 1A). No significant differences in the MB_SIR_ content compared to the control were found (*p* > 0.05).

By day 28, there were no significant changes in the samples treated with 2,4-DAPG; the BR and MB_SIR_ values were, on average, consistent with those observed on day 14. However, compared to the control group, in samples containing 2,4-DAPG (1 and 10 mg kg^−1^), the MB_SIR_ content was on average 125% higher, and the respiratory activity was 76.5% higher (*p* < 0.001).

By the end of the incubation period (day 56), CO_2_ production had sharply increased in samples containing 2,4-DAPG (1 and 10 mg kg^−1^) compared to the control. The increase was 207.9% and 142.2% higher, respectively (*p* < 0.001). A direct comparison of all treatment effects at this final time point is provided in Figure S3. During the month of the experiment, the BR increased by an average of 143% (*p* < 0.001), while the MB_SIR_ decreased by 33% (*p* < 0.05). In D-1 samples, the MB_SIR_ level was 32.4% lower than in control samples (*p* < 0.01), and the difference between D-10 samples and controls was not significant (*p* > 0.05).

On days 14 and 28, the values of QR, *q*CO_2_, and *q*CO_2_/SOC were significantly lower than the control values only in the D-10 samples (*p* < 0.05). By the end of the experiment, there was a sharp increase in D-1 samples of 359% (*p* < 0.001) and in D-10 samples of 166% (*p* < 0.01). Conversely, the MB_SIR_/SOC index on day 28 of incubation was, on average, 100% higher in samples treated with the antibiotic than in the control group (*p* < 0.001). On day 56 of the incubation period, the index decreased by 36.7% in D-1 samples (*p* < 0.001) and by 21.7% for D-10 samples (*p* < 0.05) (Table 2).

#### 3.2.2. Field Experiment

On day 7 of incubation, under the influence of 2,4-DAPG (1 and 10 mg kg^−1^) the BR (Figure 1C) and MB_SIR_ (Figure 1D) values were higher than in the control by an average of 75.2% and 25.7%, respectively (*p* < 0.001). After two weeks, CO_2_ production in D-1 samples decreased by 16.5% compared to the control group (*p* < 0.001), but still remained higher than in the control (by 57.1%, *p* < 0.05). The MB_SIR_ content continued to increase (*p* < 0.001). In contrast, the BR value in D-10 samples increased by another 34.9% (155.8% higher than in the control group, *p* < 0.005).

On day 28 of the experiment, the BR value continued to decrease in D-1 samples (by 36.3%), but remained significantly higher than in the control group, (by 38.1%). In contrast, the MB_SIR_ content decreased significantly, being 31.9% lower than in the control group (*p* < 0.001). On day 28, the BR value in the D-10 samples was 79.2% higher than in the control group, although a general trend towards decreased activity was also observed (*p* < 0.001). The MB_SIR_ content was 20.2% lower than in the control group (*p* < 0.001).

On day 56, the BR value in D-10 samples was still higher than in the control group (38.8%, *p* < 0.001), whereas in D-1 samples, it corresponded to the control level. There was no significant difference in the MB_SIR_ content between 2.4-DAPG-treated soils by the end of the experiment (*p* > 0.05), and it was, on average, 44.1% lower than in the control group (*p* < 0.001).

Throughout the incubation period, the QR, *q*CO_2_, and *q*CO_2_/SOC values in D-10 samples were significantly higher than the control values with the greatest differences occurring by the end of the experiment (*p* < 0.001). A similar trend was observed in D-1 samples (*p* < 0.005), except on day 14 of incubation, when the QR, *q*CO_2_ and *q*CO_2_/SOC values were the same as in the control group. The MB_SIR_/SOC index in samples treated with 2,4-DAPG at concentrations of 1 and 10 mg kg^−1^ was higher than the control for the first two weeks (*p* < 0.001). However, from day 28 onwards, the index values decreased, and by day 56, the average difference between the treated and control groups was 47% (*p* < 0.001) (Table 2).

### 3.3. The Effects of 2,4-DAPG on Soil Hydrolytic Enzyme Activities

#### 3.3.1. Controlled Laboratory Experiments

**Figure 2:**
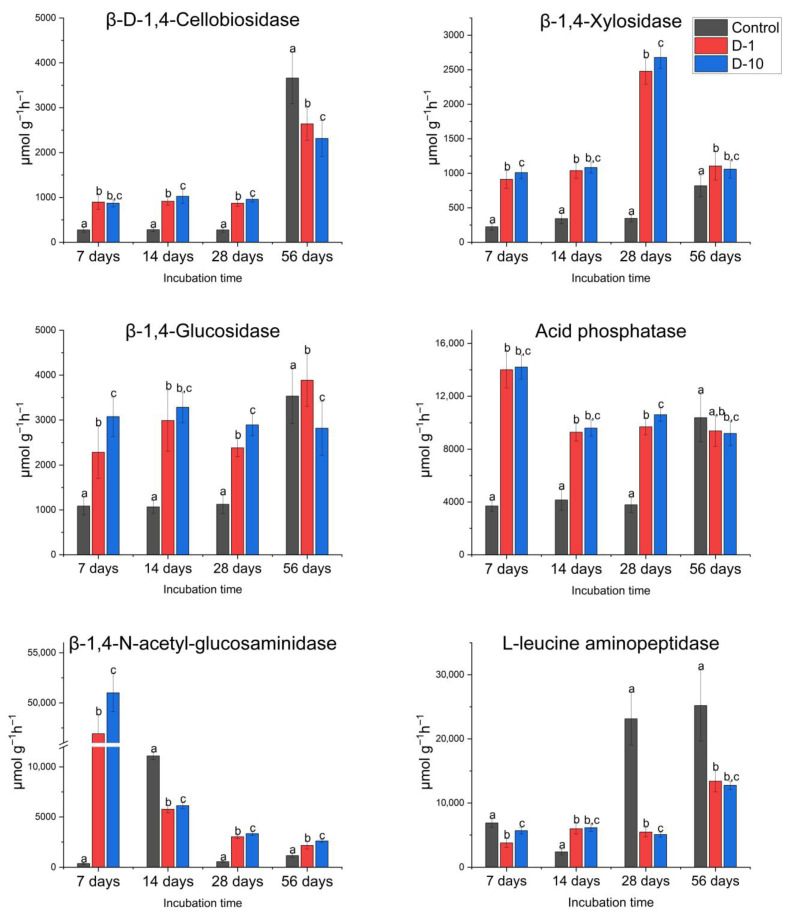
Dose-dependent effects of 2,4-DAPG on soil hydrolytic enzyme activities in controlled laboratory experiments. Enzymes associated with conversion of carbon—β-D-1,4-Cellobiosidase (CBH), β-1,4-Glucosidase (βG), β-1,4-Xylosidase (βX); nitrogen—β-1,4-N-acetyl-glucosaminidase (NAG), L-leucine aminopeptidase (LAP); phosphorus—acid phosphatase (AP). Data points represent the mean ± standard deviation from five biological replicates. Different letters indicate statistically significant differences (*p* < 0.05) based on Student’s *t*-test or Mann–Whitney U test (Holm-Bonferroni correction).

Carbon cycle. The addition of 2,4-DAPG at concentrations of 1 and 10 mg kg^−1^ to soil samples in vitro was found to significantly activate enzymes CBH, βG, and βX. This activation was observed on day 7 of the incubation (Figure 2) and was significant (*p* < 0.001). On average, the activity of these enzymes increased by 3.2-, 2.5-, and 4.3-fold for CBH, βG, and βX, respectively.

In general, the effect on the studied C-cycle enzymes was dose-dependent. However, the effect of the 2,4-DAPG treatment on the CBH enzyme was similar on day 14 of incubation (*p* > 0.5). The activity of all enzymes still exceeded control values on day 14 (by 3 times on average, *p* < 0.001). Compared to day 7, βG activity continued to increase in D-1 group (by 30.7%, *p* < 0.005) and CBH activity grew in D-10 group (by 17.6%, *p* < 0.001). The growth of βX was also observed at both concentrations of 2,4-DAPG on day 14 (D-1: 13.9%, *p* < 0.005; D-10: 7.3%, *p* < 0.01).

On the 28 th day of the incubation period, there was a significant increase in βX activity in D-1 and D-10 microcosms. On average, this was 7.4 times higher than in the control group (*p* < 0.001). Compared to day 14, enzyme activity increased by 142% (*p* < 0.005). By the end of the incubation (day 56), there was still an excess of βX and βG activity in D-1 and D-10 compared to the control group, with *p* values of less than 0.001 and 0.05, respectively. CBH and βG activity was 1.4 times lower in D-10 than in the control group, with *p* values below 0.001 (Figure 2).

Phosphorus cycle. On day 7 of the experiment, the AP activity in D-1 and D-10 microcosms was significantly higher than in the control group, with an average increase of 3.8 times (*p* < 0.001). This increase remained evident on days 14 and 28 of incubation, with an average excess of 2.3 and 2.7 times, respectively, compared to the control (*p* < 0.001), despite a slight decrease from day 7. By day 56 of incubation, the AP activity of in D-1 microcosm did not differ from the control values, while in D-10 it was lower than control values (by 11.4%; *p* < 0.05) (Figure 2).

Nitrogen cycle. Soil treatment with 2,4-DAPG at concentrations of 1 and 10 mg kg^−1^ resulted in a dose-dependent increase in NAG (*p* < 0.001) activity on day 7 of incubation (*p* < 0.001), but in a decrease in LAP activity (by 45% in D-1, *p* < 0.001 and by 17.4% in D-10, *p* < 0.001). On day 14, NAG activity decreased in both D-1 and D-10 microcosms, with values averaging 46.4% lower than the control (*p* < 0.001). LAP activity, in contrast, was higher than the control values by an average of 152.6% (*p* < 0.001).

On day 28, NAG activity in D-1 and D-10 microcosms, exceeded the control values by 5.7 times and on day56 by 2.1 times (*p* < 0.001). At the same time, LAP activity was lower than the control values: 4.4 times lower on day 28 and 1.9 times lower on day 56 of the experiment (*p* < 0.001) (Figure 2).

#### 3.3.2. Field Experiment

Carbon cycle. 2,4-DAPG significantly increased the activity of C-cycle enzymes relative to untreated controls in a dose-dependent manner. On day 14, CBH activity exceeded control values by 12.0% (D-1, *p* < 0.05) and 30.9% (D-10, *p* < 0.001), while βG activity had increased by 24.1% and 96.6% (*p* < 0.001). βX showed the most significant stimulation, surpassing control values by 46.1% and 162.7% in D-1 and D-10, respectively (*p* < 0.001). On day 56, these differences had increased further, with the treated groups demonstrating 3.3-fold (CBH), 4.9-fold (βG), and 10.5-fold (βX) higher activity compared to the control groups.

**Figure 3:**
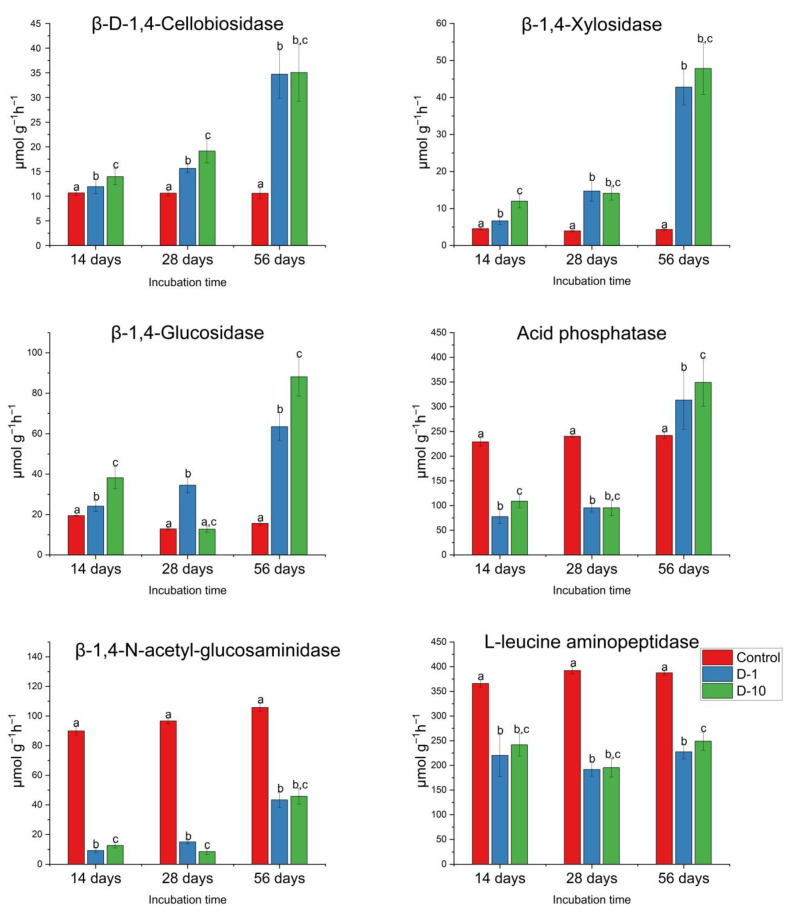
Dose-dependent effects of 2,4-DAPG on soil hydrolytic enzyme activities in field experiments. Data points represent the mean ± standard deviation from five biological replicates. Different letters indicate statistically significant differences (*p* < 0.05) based on Student’s *t*-test or Mann–Whitney U test (Holm-Bonferroni correction).

The increase in activity continued on day 28 (*p* < 0.05). On day 56, the CBH activity in D-1 and D-10 groups was on average 3.3 times higher (*p* < 0.05) than in the control group. The βX value was 10.5 times higher (*p* < 0.001), and the βG value was 4.9 times higher (*p* < 0.001) (Figure 3).

Phosphorus cycle. During the first month of incubation (days 14 and 28), the AP activity in D-1 and D-10 microcosms was less than half that of the control. However, by day 56, the enzyme activity had increased by 129.5% in D-1 (*p* < 0.01) and 144% in D-10 (*p* < 0.001) compared to the control (Figure 3).

Nitrogen cycle. Throughout the incubation period, the activity of NAG and LAP enzymes in soil samples treated with 2,4-DAPG at concentrations of 1 and 10 mg kg^−1^ was significantly lower (*p* < 0.05) than in the control group. Specifically, NAG activity was, on average, by 87.1% (*p* < 0.05) lower in D-1 sample and by 88.5% (*p* < 0.001) lower in D-10 sample than in the control after 28 days.

On day 56 of the incubation period, NAG activity in the treated samples had increased compared to day 28 levels. In D-1 sample, it increased 2.9-fold (*p* < 0.001), and in D-10 sample it increased 5.4-fold (*p* < 0.005). Despite this increase, however, NAG activity remained below control values, by an average of 57.9%, in both samples (*p* < 0.05). LAP activity was suppressed throughout the 56-day incubation period under 2,4-DAPG treatment (Figure 3). On day 14, activity had decreased by 39.8% (D-1) and 34% (D-10) compared to the control group (*p* < 0.001), with further reductions of 13% (D-1, *p* < 0.05) and 19.1% (D-10, *p* < 0.001) by day 28. Overall, LAP activity did not change significantly throughout the incubation period, remaining lower than the control value in the experimental microcosms (*p* > 0.40) (Figure 3).

### 3.4. Environmental Modulation of Microbial Community Function

Comprehensive correlation analysis revealed significant environmental influences on microbial community activity (Figure S4). Precipitation during the preceding week strongly correlated with basal respiration (Rs = 0.67, *p* < 0.05), indicating enhanced microbial metabolic activity following rainfall events. Conversely, average temperature showed significant negative correlations with cellulolytic enzyme activities (CBH: Rs = −0.58; BG: Rs = −0.53), suggesting thermal suppression of some carbon-cycling functions. Notably, microbial biomass maintained relatively stable relationships with environmental factors.

### 3.5. Diversity and Composition of Bacterial Soil Community

**Figure 4:**
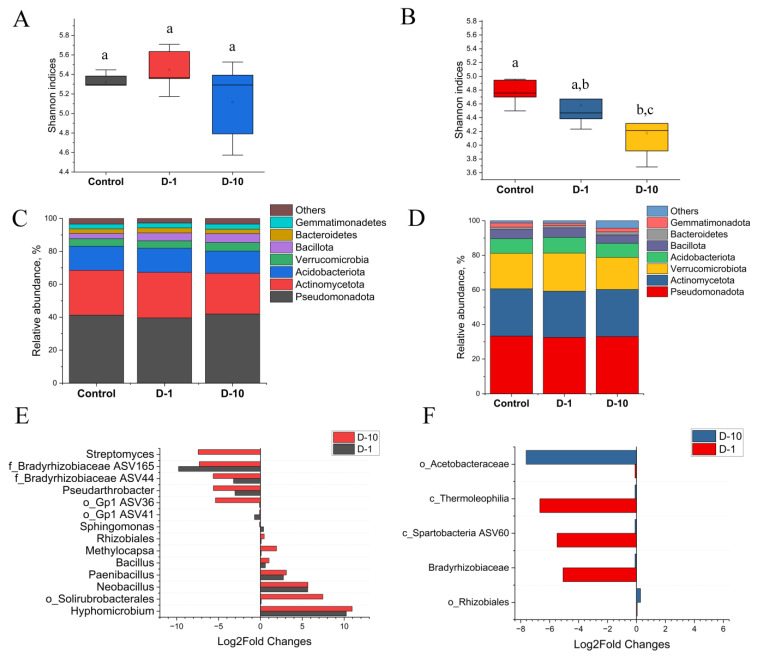
The diversity and composition of soil bacterial communities exposed to 2,4-DAPG are presented in this study. The data for laboratory trials (**A**,**C**,**E**) and field trials (**B**,**D**,**F**) are included. Alpha diversity of the communities in the laboratory (**A**) and the field (**B**) is presented (different letters indicate statistically significant differences, one-way ANOVA, Tukey’s test, *p* < 0.05). The taxonomic composition at the phylum level (**C**,**D**) is presented. Differences in the abundance of ASVs belonging to bacterial genera between control and 2,4-DAPG-treated microcosms were expressed as log2fold change values, and only dominant ASVs with significant differences (*p* < 0.05, Mann–Whitney U test, Holm-Bonferroni correction) are presented between compared microcosm (**E**,**F**).

The alpha diversity of the bacterial community under laboratory conditions was assessed using the Shannon index and ranged from 5.31 ± 0.1 (Control) to 5.11 ± 0.4 (D-10) (Figure 4A). The Chao1 index ranged from 311 ± 43.7 (Control) to 270 ± 108 (D-10). However, these differences between treatments were not statistically significant (*p* > 0.05).

By contrast, under field conditions, 2,4-DAPG reduced the Shannon index in a dose-dependent manner from 4.77 ± 0.19 (control) to 4.17 ± 0.4 (D-10; *p* < 0.05; Figure 4B). In comparison, the Chao1 index showed no significant change, varying from 182 ± 32.9 to 103 ± 41.9 (*p* > 0.05; Figure 4B).

At the phylum level, the bacterial community in the laboratory microcosms on day 28 was dominated by Pseudomonadota, Actinomycetota, Acidobacteriota, Verrucomicrobiota, Bacillota, and Bacteroidota. The taxonomic composition was altered by 2,4-DAPG. Most notably, the relative abundance of Bacillota increased in a dose-dependent manner from 3.18 ± 0.36% in the control group to 4.7 ± 0.31% in D-1 and 5.5 ± 0.64% in D-10 (*p* < 0.05). Significant changes were also observed for other phyla in D-10 treatment: Acidobacteriota decreased from 14.62 ± 0.5% to 13.6 ± 0.83% (*p* < 0.05), Actinomycetota decreased from 27.22 ± 3.17% to 24.97 ± 2.48% (*p* < 0.05), and Verrucomicrobiota increased from 4.57 ± 0.56% to 5.24 ± 0.46% (*p* < 0.05) (Figure 4C).

The same phyla were dominant in field conditions. However, in contrast to the laboratory experiment, no significant differences in bacterial community composition at the phylum level were observed between treatments (Figure 4D).

The impact of 2,4-DAPG on the relative abundance of bacterial ASVs was more pronounced in the laboratory than in the field. On day 28, exposure to 1 mg kg^−1^ and 10 mg kg^−1^ of 2,4-DAPG altered the abundance of 5.98% and 8.53% of all ASVs in laboratory microcosms, respectively. In contrast, under field conditions, the same concentrations affected a smaller proportion of ASVs: 5.21% and 3.89%, respectively.

Analysis of the dominant genera revealed that the abundance of 15 ASVs had changed significantly (Figure 4E; full list in Table S1). Taxa with increased abundance: In D-1 treatment, *Hyphomicrobium* (Log_2_FC +10.3, *p* < 0.05), *Neobacillus* (Log_2_FC +5.65, *p* < 0.05), and *Paenibacillus* (Log_2_FC +2.8, *p*< 0.05) were enriched. In D-10 treatment, the aforementioned taxa were also enriched, along with o_Solirubrobacterales (Log_2_FC +7.45, *p* < 0.05), *Methylocapsa* (Log_2_FC +1.98, *p* < 0.05), and *Bacillus* ASV18 (Log_2_FC +1.03, *p* < 0.05).

Taxa with decreased abundance: In D-1 treatment, f_*Bradyrhizobiaceae* ASV165 (Log_2_FC −9.77, *p* < 0.05), f_*Bradyrhizobiaceae* ASV44 (Log_2_FC −3.21, *p* < 0.05), and *Pseudarthrobacter* (Log_2_FC −3.03, *p* < 0.05) were suppressed. D-10 treatment resulted in a stronger suppression of these taxa and additionally reduced the abundance of *Streptomyces* (Log_2_FC −7.47, *p* < 0.05) and o_Gp1 (Log_2_FC −5.37, *p* < 0.05).

Field Experiment. The effect of 2,4-DAPG was considerably weaker, with only 5 dominant ASVs significantly affected (Figure 4F). A significant decrease in abundance was observed for c_Thermoleophilia (Log_2_FC −6.67, *p* < 0.05), c_Spartobacteria ASV60 (Log_2_FC −5.48, *p* < 0.05), and f_*Bradyrhizobiaceae* (Log_2_FC −5.07, *p* < 0.05) in D-1 treatment, and for f_*Acetobacteriaceae* (Log_2_FC −7.62, *p* < 0.05) in D-10 treatment.

A minor but significant increase was noted for o_Rhizobiales (Log_2_FC +0.28, *p* < 0.05) in the D-1 microcosms. A complete list of all ASVs that showed statistically significant changes in abundance is provided in Table S1.

### 3.6. Diversity and Composition of Fungal Soil Community

**Figure 5:**
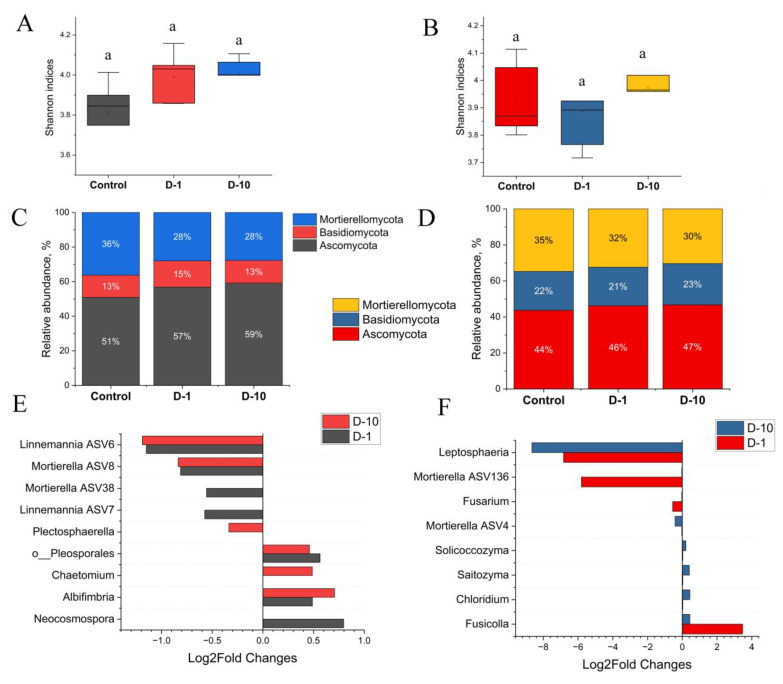
The diversity and composition of soil fungal communities when exposed to 2,4-DAPG. The data are presented for the results of laboratory trials (**A**,**C**,**E**) and field trials (**B**,**D**,**F**). The alpha diversity of fungal communities in the laboratory (**A**) and the field (**B**) is presented (different letters indicate statistically significant differences, one-way ANOVA, Tukey’s test, *p* < 0.05). The taxonomic composition of soil microcosms at phylum level (**C**,**D**). Differences in the abundance of ASVs belonging to fungal genera between control and 2,4-DAPG-treated microcosms were expressed as log_2_fold change values, and only dominant ASVs with significant differences (*p* < 0.05, Mann–Whitney U test, Holm-Bonferroni correction) are presented between compared microcosm (**E**,**F**).

The alpha diversity of the fungal community responded differently to 2,4-DAPG under laboratory and field conditions. In the laboratory experiment, an increasing trend in fungal alpha diversity was observed. The Shannon index augmented from 3.81 ± 0.17 in the control group to 3.99 ± 0.13 (D-1) and 4.01 ± 0.1 (D-10) (Figure 5A). Similarly, the Chao1 index increased from 177 ± 38 to 216 ± 40 and 217 ± 18.2, respectively. However, these changes were not statistically significant (*p* > 0.05).

Under field conditions, the addition of 2,4-DAPG had no significant impact on fungal alpha diversity (*p* > 0.05). The Shannon indices remained stable across all treatments: 3.93 ± 0.14 (control), 3.87 ± 0.16 (D-1), and 3.97 ± 0.1 (D-10) (Figure 5B). The Chao1 index exhibited greater variability, but also was not statistically significant, with values of 145 ± 29 (control), 120 ± 34 (D-1), and 133 ± 29 (D-10) (*p* <0.05).

Exposure to 2,4-DAPG in laboratory on day 28 significantly altered the composition of the fungal community at the phylum level, increasing the relative abundance of Ascomycota and Basidiomycota, and reducing the proportion of Mucoromycota (Figure 5C).

A dose-dependent response was observed at the genus level (Figure 5E; full data in the Table S2). The treatment altered the abundance of 5.52% and 6.91% of all fungal ASVs at concentrations of 1 mg kg^−1^ (D-1) and 10 mg kg^−1^ (D-10), respectively. The higher dose (D-10) had a more pronounced stimulatory effect, with the majority of affected ASVs (5.53% of the total) showing increased abundance.

Taxa with increased abundance: Several genera were consistently enriched across treatments, including *Albifimbria* ASV42, o_*Pleosporales* ASV32, and *Neocosmospora* (in D-1). The D-10 treatment specifically enriched *Chaetomium*.

Taxa with decreased abundance: Key genera within Mucoromycota were consistently suppressed, including as *Linnemannia* ASV6 (Log_2_FC −1.15, *p* < 0.05) and *Mortierella* ASV8 (Log_2_FC −0.81, *p* < 0.05) in both treatments.

By contrast, field conditions mitigated the impact of 2,4-DAPG. A smaller proportion of ASVs was affected: 4.11% at D-1 and 4.23% at D-10. Furthermore, the effect was primarily inhibitory at the lower dose (D-1), where the abundance of 3.31% ASVs decreased.

The most significant changes involved a sharp decrease in the abundance of several taxa, including f_*Mortierellaceae* (Log_2_FC −7.22, *p* < 0.05), *Leptosphaeria* ASV100 (Log_2_FC from −6.83 to −8.65, *p* < 0.05), and *Mortierella* ASV136 (Log_2_FC −5.80, *p* < 0.05) across various treatments (Figure 5F). A limited stimulatory effect was observed for genera such as *Chloridium*, *Saitozyma*, and *Solicoccozyma* at the D-10 concentration.

In summary, the application of 2,4-DAPG consistently suppressed fungi from the Mucoromycota phylum (e.g., *Mortierella*, *Linnemannia*) in both experiments. However, the overall impact on fungal communities was markedly reduced in the field, where the diversity of responsive taxa was lower.

### 3.7. Analysis of the Ecological Network Structure of Bacterial and Fungal Communities

**Figure 6:**
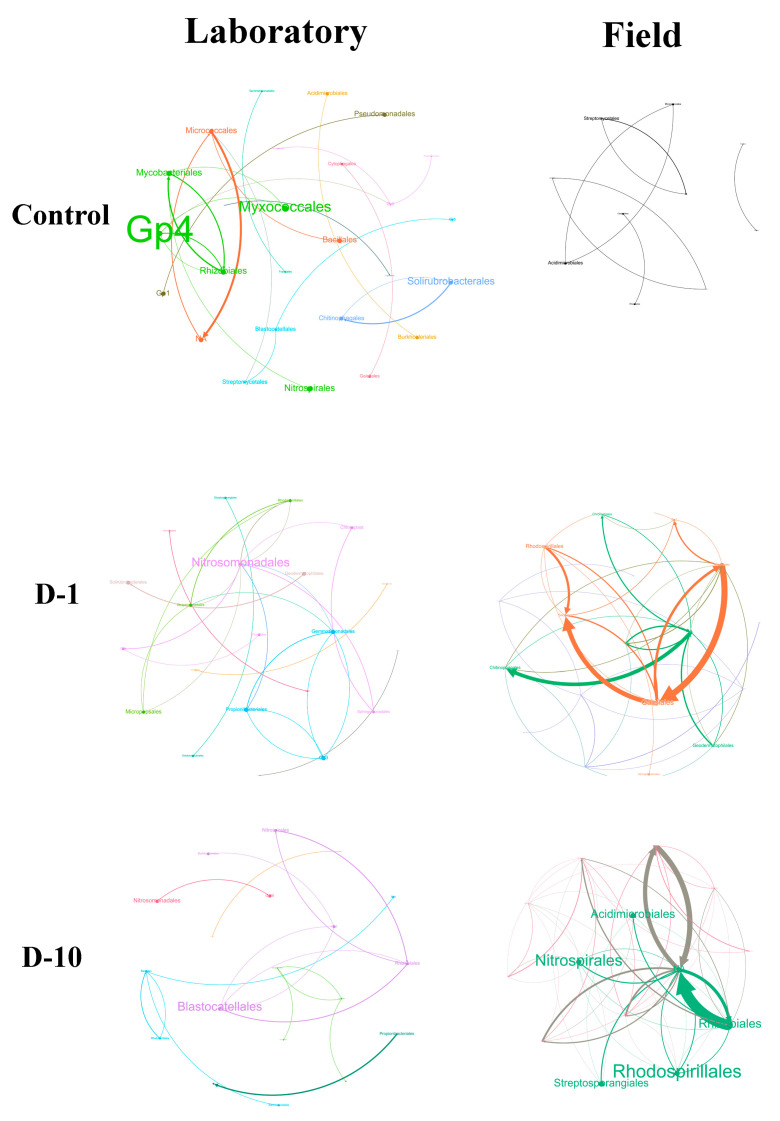
Bacterial co-occurrence networks under 2,4-DAPG exposure (1 and 10 mg kg^−1^) in laboratory and field trials. Nodes represent ASVs at the genus level (size reflects connectivity degree), and colors indicate network modules. The size of the genus label font is proportional to the node’s relative importance as a network hub.

The addition of 2,4-DAPG produced contrasting structural changes in bacterial co-occurrence networks under laboratory and field conditions (Figure 6, Tables S3–S6; See Appendix A for an explanation of topological properties of the networks).

Under laboratory conditions, bacterial networks demonstrated significant simplification and increased centralization in response to 2,4-DAPG. At a concentration of 1 mg kg^−1^, the bacterial network underwent substantial simplification, as evidenced by a reduction in the number of nodes (taxa) from 61 in the control to 36. This was accompanied by a significant increase in network complexity and non-random structure, as follows from an increase in the average clustering coefficient (avgCC) from 0 to 0.236 and in centralization (CD) from 0.094 to 0.334. Concurrently, we observed an increase in the relative abundance of the order *Nitrosomonadales.* At a concentration of 10 mg kg^−1^, further simplification occurred (37 nodes), with a shift in dominance to *Blastocatellales*, a restored power-law distribution (R^2^ = 0.976), and reduced maximum connectivity (from 13 to 6).

By striking contrast, field conditions prompted a significant increase in network complexity. At 1 mg kg^−1^, the number of nodes and connections augmented from 14 to 67 and from 9 to 147, respectively, with *Rhizobiales* and *Gaiellales* emerging as central hubs (max degree = 12). At 10 mg kg^−1^, the network became denser (429 connections, density = 0.289), exhibiting high average connectivity (avgK = 15.6) and new dominant nodes (*Rhodospirillales*, max degree = 28). However, the disruption of the power law (R^2^ = 0.032) and the decreased efficiency (E = 0.626 compared to 0.821 in the control group) suggests a loss of stability and an increase in randomness.

**Figure 7:**
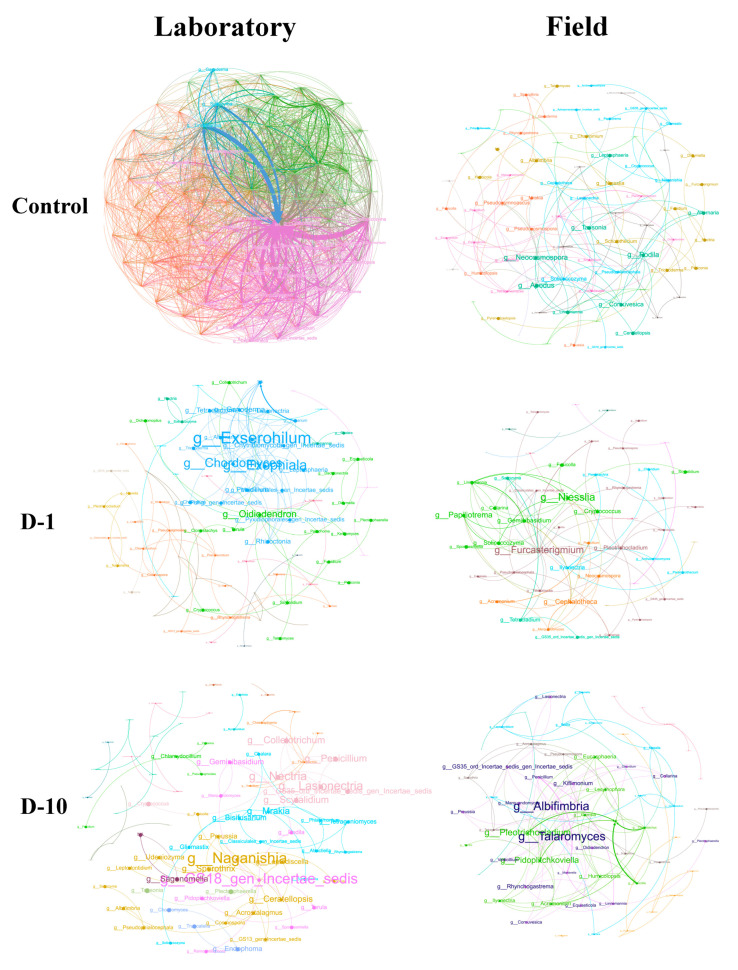
Fungal co-occurrence networks under 2.4-DAPG exposure (1 and 10 mg kg^−1^) in laboratory and field trials. Nodes represent ASVs at the genus level (size reflects connectivity degree), and colors indicate network modules. The size of the genus label font is proportional to the node’s relative importance as a network hub.

In the laboratory, fungal networks exhibited a strong, dose-dependent response to 2,4-DAPG (Figure 7, Table S5). They shifted from a highly connected structure (150 nodes, 3055 connections, avgK = 40.7, density = 0.273) to a fragmented and simplified state (82–90 nodes, 84–130 connections, avgK = 2.0–2.9, density = 0.025–0.032).

Dominance *Neocosmospora* (max degree = 85, control) was shifted first to *Exserohilum* (1 mg kg^−1^) and then to *Naganishia* (10 mg kg^−1^). The latter assumed a central role across all topological metrics (betweenness = 169, eigenvector centrality = 0.531).

Conversely, the structural integrity of fungal networks in the field was maintained (Figure 7, Table S6), with node counts of 80 → 61 → 78 and connections of 130 → 93 → 137 across the concentration gradient. Key properties such as clustering (avgCC: 0.196 → 0.240) were preserved or enhanced, and new dominant taxa emerged (*Furcasterigmium* at 1 mg kg^−1^, *Pleotrichocladium* at 10 mg kg^−1^).

## 4. Discussion

### 4.1. Biochemical and Ecological Drivers of Microbial Community Responses to 2,4-DAPG

2,4-DAPG is a broad-spectrum antibiotic produced by pseudomonads that has been widely studied for its role in the biological control of plant pathogens [12,29]. Our study reveals that the antimicrobial activity of 2,4-DAPG in a complex soil environment is not a simple function of its dose, but is profoundly filtered by the ecological context. This work provides several key advances beyond the previous, largely pathogen-focused literature on this compound [1,4,5,6,7,8,9].

First, we demonstrate that the impact observed in controlled laboratory microcosms is a poor predictor of the effect in a natural field setting, where functional redundancy and community buffering capacity attenuate the disruption. Second, we document a divergent response between kingdoms, where an antimicrobial compound can inadvertently confer a competitive advantage to specific fungi, potentially shifting soil ecological balances. Finally, by analyzing eco-physiological indices and co-occurrence networks, we provide a mechanistic basis for these observations, linking the compound’s action to changes in microbial metabolism and community architecture.

The profound restructuring of the soil microbiome observed in our study arises from a complex interaction between the biomolecular activity of the compound and its ecological context. 2,4-DAPG, an amphipathic polyphenolic compound, primarily acts by rapidly permeabilizing biological membranes [12], which generally makes Gram-positive bacteria more susceptible. However, the striking enrichment of specific Bacillota, such as *Bacillus* and *Paenibacillus*, highlights a critical exception. This suggests strong selection for resistant taxa with specialized adaptations such as enhanced cell envelope fortification or efficient efflux pumps. The suppression of Actinobacteria (e.g., *Streptomyces*) and Pseudomonadota (e.g., Bradyrhizobiaceae) aligns with their documented vulnerability [30]. This demonstrates how intrinsic cellular differences cascade into community-level taxonomic shifts. These shifts are reflected in altered metabolic quotients, indicating a long-term metabolic adaptation to energetic stress.

Critically, the production and impact of 2,4-DAPG are not static but are dynamically regulated by the biotic environment. Its synthesis in pseudomonads can be triggered by inter-bacterial competition via direct cell-to-cell contact (e.g., with *Lysobacter*) and in response to eukaryotic predators like amoebae [31,32]. Furthermore, the concentration and antifungal efficacy of 2,4-DAPG have been shown to increase with the diversity of the producing *Pseudomonas* community itself [33]. This ecological framework is essential for interpreting our results: the attenuated effects in the field likely reflect a scenario where the natural diversity of the microbial community modulates both the production and the functional impact of the antibiotic, buffering its disruptive potential compared to the simplified laboratory system.

Beyond direct toxicity, our results are consistent with the sub-inhibitory, signaling role of 2,4-DAPG. The observed suppression of certain bacterial or fungal functions aligns with its capacity to inhibit quorum sensing in bacteria and fungi [12,34]. This interference with microbial communication could disrupt the cooperative organization of the soil community, contributing to the simplified co-occurrence networks we detected in laboratory microcosms.

The divergent response of the fungal community is particularly revealing. Our data, along with the results of a previous study, indicates that 2,4-DAPG triggers specific molecular responses in different taxa [35]. In saprotrophic fungi, it induces a coordinated stress response aimed at maintaining metabolic homeostasis, including the dramatic upregulation of genes for carbohydrate metabolism and hydrolytic enzymes. This aligns perfectly with our observed increase in fungal alpha diversity and sustained stimulation of cellulolytic (CBH, βG) and chitinolytic (NAG) activities in the lab. The antibiotic stress appears to trigger a survival strategy centered on the hyper-production of energy-yielding enzymes. This fundamental difference in transcriptional response likely underlies the competitive advantage conferred to saprotrophic fungi like *Chaetomium* and *Naganishia*, which we observed dominating the treated communities.

In conclusion, the ecological patterns we documented are emergent properties of a multi-layered interaction network. They are driven not only by the direct membrane-disrupting toxicity of 2,4-DAPG but also by its role as a chemical messenger, its production being modulated by biotic interactions, and its ability to trigger taxon-specific transcriptional reprogramming that ultimately determines the fitness and functional output of different microbial groups.

### 4.2. Dose-Dependent Effects on Microbial Activity

The functional response of the soil microbiome to 2,4-DAPG reveals a fundamental dichotomy. At low concentrations, it acts primarily as a resource. However, at high concentrations, it imposes a toxic stress. The manifestation of these roles is heavily dependent on environmental context. We have found that 2,4-DAPG modulates the function of soil microbial communities, with the effects varying depending on the dose and incubation time. The D-1 treatment resulted in significant stimulation of BR and MB_SIR_ (D-1 > D-10 > control), which indicates that 2,4-DAPG serves as an easily accessible substrate in the soil matrix.

2,4-DAPG is a polyphenolic molecule with two acetyl groups (-COCH_3_) at positions 2 and 4 of the phloroglucinol ring. Therefore, at a dose of 1 mg kg^−1^, this substance acts as a source of carbon and energy. Studies have shown that the breakdown of 2,4-DAPG, catalyzed by the PhlG (C-C bond hydrolase), promotes bacterial growth under conditions of carbon/nitrogen deprivation [36]. Microorganisms rapidly utilize it, resulting in an increase in metabolic activity (↑QR) and a decrease in metabolic efficiency (↑*q*CO_2_); while carbon availability stimulates microbial biomass growth (↑MB_SIR_, ↑MB_SIR_/SOC). In the case of a high dose (10 mg kg^−1^), “2,4-DAPG = resource”, but its uptake is limited by the metabolic costs of adaptation. The results from the following three weeks (days 14 and 28) show that the microbial community in D-1 used the available substrate and returned to initial levels.

In D-10 microcosms, stable respiration and a still high microbial biomass content (although reduced relative to Day 7 of incubation), on the one hand, indicate maximum efficiency in the utilization of 2,4-DAPG. On the other hand, this apparent “well-being” may actually be a state of latent stress (↓QR, ↓*q*CO_2_) caused by changes in the microbial community structure (decreased bacterial diversity and increased fungal diversity) and its adaptation to the stress factor. The concurrent rise in biomass carbon (↑MB_SIR_/SOC) and fall in the metabolic quotient (↓*q*CO_2_/SOC), indicate that high doses of 2,4-DAPG doses favor the accumulation of resistant, but metabolically slow microorganisms.

By day 56 of the experiment, following the depletion of 2,4-DAPG reserves, the microbial communities in treatments with different doses of antibiotics showed signs of significant disruption to their functioning. However, the mechanisms behind this disruption differed between the treatments. In D-1 microcosms, the depletion of the antibiotic substrate forced the community to transition to the use of native soil organic compounds, some of which are difficult to decompose.

This process requires high energy costs per unit of biomass and has low metabolic efficiency. This explains the sharp increase in basal respiration (BR↑). In oligotrophic communities, most of the energy from the consumed substrate is used to maintain cellular functions under conditions of resource scarcity rather than for growth. This is confirmed by a significant decrease in microbial biomass (MB_SIR_↓) [37,38,39].

In D-10 microcosms, an increase in BR and a decrease in microbial biomass are markers of chronic stress caused by long-term exposure to high doses of antibiotics. This contributes to the following: (a) structural reorganization of the microbial community (a shift in composition), (b) accumulation of dead microbial cells (an additional source of bioavailable carbon), (c) increased energy costs for maintaining homeostasis under toxic conditions, (d) metabolic costs of antibiotic resistance, and e) the synthesis of enzymes [40].

A sharp increase in QR, *q*CO_2_ and *q*CO_2_/SOC, coupled with a simultaneous decrease in the MB_SIR_/SOC at both doses on day 56, confirms a significant disturbance to the functional state of the soil microbial community. However, depending on the antibiotic dose, the patterns of response and underlying mechanisms, such as substrate depletion and transition to complex organic matter in D-1 versus chronic toxic stress and restructuring in D-10, differ significantly.

The results of the field experiment are in good agreement with the laboratory data. 2,4-DAPG can function either as an easily accessible carbon source that stimulates short-term metabolic activity (breathing burst), or as a stress factor that requires additional energy expenditure. However, it is important to note that the field experiment is a semi-open system influenced by various factors, such as temperature, humidity, and precipitation.

Under these conditions, the effect of 2,4-DAPG on the D-1 variant was short-lived, persisting only until day 28. The stimulated microbial communities likely utilized not only the introduced 2,4-DAPG but also triggered a strong positive priming effect. This leads to the intensive decomposition of the labile fraction of native soil organic carbon (SOC), as indicated by the decrease observed in Table 1. In the case of the higher dose (D-10), the stress/stimulation effect was more pronounced and prolonged (lasting up to 56 days), requiring additional energy expenditure for detoxification and adaptation (as reflected by increased metabolic quotients: D-10 ↑QR, ↑*q*CO_2_ > D-1).

The reduction in microbial biomass observed in the treated samples by the end of the experiment can be attributed to the depletion of readily available organic matter, as well as a shift in the metabolic strategy of the adapted community. This shift is characterized by decreased carbon use efficiency (as evidenced by D-10 ↓MB_SIR_/SOC, ↑*q*CO_2_/SOC > D-1).

Thus, 2,4-DAPG acts as a dose-dependent regulator of the soil microbial community, serving either as a short-term nutrient source or as a long-term stress factor. The intensity, duration, and consequences of its influence depend significantly on environmental circumstances. Under controlled laboratory environment (stable conditions), the impact of 2,4-DAPG is significant and pronounced, resulting in long-lasting alterations to the performance of the soil microbiome. In contrast, in a field experiment involving dynamic abiotic variables, its influence is reduced and attenuated, likely due to the buffering capacity of the soil ecosystem.

### 4.3. Functional Shifts in Nutrient Cycling: Enzyme Responses

In this section, we analyze the dose-dependent effects of C, N, and P cycle enzymes on soil fertility and nutrient availability.

In the carbon cycle, a pronounced and sustained stimulation of cellulolytic enzyme activity (CBH, βG, βX) was observed. The sharp initial increase, which peaked at 7.4-fold on day 28, indicates a long-term enhancement of hemicellulose degradation. This aligns with the hypothesis that 2,4-DAPG or its decomposition products served as a priming agent, stimulating microbial communities to depolymerize complex soil organic matter. From an ecological perspective, this suggests potential acceleration of the decomposition of labile soil organic matter, which could temporarily boost microbial activity but poses a risk to long-term soil carbon storage if sustained. The subsequent decline in activity below control levels in D-10 treatment on day 56 likely reflects substrate depletion (e.g., of the easily available C fraction) and/or a fundamental adaptive restructuring of the microbial community towards alternative functions. This is consistent with our observed changes in microbial biomass and respiration.

The response of the phosphorus cycle, mediated by alkaline phosphatase activity, was transient. The initial 3.8-fold increase suggests a rapid microbial demand for inorganic P, potentially to support accelerated growth and enzyme production triggered by the antibiotic amendment. This pulsed mineralization could lead to a temporary, unsynchronized flush of available phosphorus, potentially affecting plant phosphorus uptake efficiency. The subsequent decline to levels below those of the control in D-10 treatment by the end of the experiment could be attributed to a combination of factors, such as exhaustion of labile organic P pools, a shift in microbial community structure away from efficient P-solubilizers, or a general inhibition of microbial activity under prolonged exposure to high-dose stress.

The most complex, dose-dependent effects were observed in the nitrogen cycle. The early suppression of LAP activity coupled with the stimulation of NAG indicates a redirection of microbial nutrient acquisition strategies from protein degradation (proteolysis) towards chitin decomposition. The 5.7-fold increase in NAG activity on day 28 strongly implies an activation of the fungal community. This shift suggests a fundamental reconfiguration of the N cycle, potentially altering the timing and forms of plant-available nitrogen by favoring chitin-derived nitrogen over protein-derived nitrogen, which could influence crop nitrogen use efficiency. The persistent suppression of LAP suggests a reduced role for bacteria in proteolysis, possibly due to direct inhibition by 2,4-DAPG or competitive exclusion by stimulated fungi. On day 56, the widespread decrease in the activity of most enzymes in the high-dose treatment (D-10) suggests two possible explanations: (1) The potential toxicity of antibiotic; or (2) the completed adaptive restructuring of microbiota towards a new functional state that prioritizes survival over decomposition.

In conclusion, 2,4-DAPG functions as a potent modulator of soil enzyme activity. Its initial role appears to be that of a biochemical catalyst, initiating a burst of decomposing activity. However, its long-term effect transforms it into a driving force for selecting functional changes in the microbiome. The final outcome for soil fertility—whether short-term stimulation or long-term inhibition of biogeochemical cycles—depends on the interaction between the dose, duration of exposure, and the resilience of the microbial community. The contrast between the strong laboratory effects and the weaker field responses highlighted in our study suggests that, in real agricultural systems, the redundancy of soil microorganisms likely protects against significant long-term disruptions to nutrient cycling.

### 4.4. Structural Reorganization of Bacterial and Fungal Communities

This subsection compares the taxonomic and diversity responses of bacteria and fungi to 2,4-DAPG exposure, highlighting differences between these two kingdoms. We have demonstrated that 2,4-DAPG acts as a selective force on soil microbiomes, with its effects being attenuated in field conditions. This study demonstrates that 2,4-DAPG acts as a selective force on soil microbiomes, the effects of which are reduced under field conditions. Significant compositional shifts were observed, including an increase in the abundance of Bacillota (e.g., *Bacillus*, *Neobacillus*, *Paenibacillus*) and a decrease in the abundance of Actinomycetota (e.g., *Streptomyces*) and symbiotic *Bradyrhizobiaceae* [41,42]. This suggests a potential disruption to decomposition of organic matter and nitrogen fixation.

Bacterial sensitivity was markedly lower in the field (4% of ASVs responded) than in the laboratory (8.5% at 10 mg kg^−1^). This is attributed to physiological adaptations (e.g., dormancy in Bacillota; [43]) and environmental buffering via complex trophic networks [44]. Fungal responses were also context-dependent (e.g., *Linnemannia* resistance in the field), likely due to stress-tolerant structures or symbiosis [45,46,47].

The responses of fungi and bacteria diverged: fungal alpha diversity was stable, suggesting tolerance or utilization of 2,4-DAPG (e.g., by *Chaetomium*), while bacterial diversity decreased in the field conditions. High doses inhibited bacteria but benefited fungi (5.53% ASVs), which is consistent with meta-analyses showing decreased bacterial/fungal ratios under antibiotic treatments [48].

Our analysis of microbial co-occurrence networks revealed that 2,4-DAPG fundamentally restructured soil community organization. In controlled laboratory conditions, the antibiotic simplified bacterial networks by reducing species richness and interaction density, while disrupting the connectivity of fungal networks. This indicates a loss of functional redundancy and cooperative potential, making the community more vulnerable to additional stressors.

In contrast, in field conditions, the bacterial network maintained greater complexity, and the fungal network preserved its structural integrity. These findings suggest that the sustained connectivity of these networks is crucial for soil resilience. It ensures the persistence of multiple pathways for nutrient cycling and energy flow, which are essential for maintaining ecosystem function. The preservation of complex interaction networks in natural soils underscores their inherent capacity to buffer anthropogenic disturbances.

Thus, the “pure” antibiotic effect observed in the laboratory is significantly altered in the field due to pre-adaptation to stress, functional redundancy, and horizontal gene transfer [40]. It is risky to extrapolate the laboratory results to natural ecosystems; fungal persistence and bacterial network variability highlight interkingdom differences in stress response strategies.

The markedly attenuated response of the soil microbiome to 2,4-DAPG in the field compared to laboratory microcosms can be attributed to several key ecological factors inherent in natural ecosystems. Firstly, field communities are pre-adapted to a wider range of abiotic stresses that select for general stress response traits, such as sporulation and efflux pumps, which provide cross-tolerance to antibiotics. Secondly, higher functional redundancy and network complexity in the field ensure that key processes like antibiotic degradation can continue even if some taxa are inhibited. Lastly, the open nature of field systems facilitates horizontal gene transfer, allowing for rapid community-wide adaptation through the mobilization of catabolic genes from the soil resistome [30]. In contrast, the stable and closed environment of the laboratory microcosm supports a less pre-adapted community with a lower functional buffering capacity.

## 5. Conclusions

In conclusion, the data obtained emphasize that the actual ecological impact of antibiotics differs significantly from the laboratory models due to the complex interactions between species and adaptation mechanisms within soil communities. These factors must be considered when evaluating the ecological risks associated with the use of antimicrobial compounds in agriculture. At the same time, assessing the effect of these compounds on soil communities also requires consideration of interkingdom interactions, as well as the fundamentally different adaptation strategies employed by bacteria and fungi.

These findings are important for understanding the potential consequences of using 2,4-DAPG in agroecosystems. The bacterial-to-fungal population ratio in the microbiota has the potential to have cascading effects on soil health and plant productivity, underlining the importance of carefully considering these interactions.

## Figures and Tables

**Table 1:** Soil organic carbon (SOC) content was measured in g kg^−1^, treated with different concentrations of 2,4-DAPG in both laboratory and field experiments. Each biological replicate consisted of three analytical samples.

Microcosms	Laboratory				Field			
	Incubation Time, Days							
	7	14	28	56	7	14	28	56
Control	17.4 ± 0.69 ^a^	17.5 ± 1.09 ^a^	17.4 ± 0.53 ^a^	17.3 ± 1.13 ^a^	15.5 ± 0.58 ^a^	15.5 ± 0.43 ^a^	15.7 ± 1.36 ^a^	15.0 ± 0.69 ^a^
D-1	18.9 ± 1.07 ^b^	19.1 ± 0.69 ^b^	18.7 ± 0.78 ^b^	18.4 ± 0.76 ^b^	13.4 ± 0.43 ^b^	16.5 ± 1.07 ^b^	15.8 ± 0.60 ^a^	15.6 ± 0.82 ^b^
D-10	18.9 ± 0.93 ^b,c^	19.1 ± 0.59 ^b,c^	19.8 ± 0.63 ^c^	18.3 ± 0.75 ^b,c^	14.7 ± 1.01 ^c^	14.7 ± 1.03 ^c^	16.2 ± 0.69 ^a^	16.0 ± 0.50 ^b,c^

Note: Different letters indicate statistically significant differences (*p* < 0.05) based on Student’s *t*-test or Mann–Whitney U test (Holm-Bonferroni correction) (as appropriate see Section 2.7).

**Table 2:** Calculated indicators of ecophysiological activity of the microbial community in soils treated with different concentrations of 2,4-DAPG.

Microcosms	Laboratory Conditions				Field Conditions			
	Incubation Time, Days							
	7	14	28	56	7	14	28	56
	QR							
Control	0.1 ± 0.01 ^a^	0.16 ± 0.05 ^a^	0.19 ± 0.08 ^a^	0.14 ± 0.04 ^a^	0.07 ± 0.01 ^a^	0.06 ± 0.02 ^a^	0.03 ± 0.01 ^a^	0.09 ± 0.04 ^a^
D-1	0.13 ± 0.05 ^b^	0.14 ± 0.04 ^a,b^	0.15 ± 0.02 ^a,b^	0.67 ± 0.37 ^b^	0.10 ± 0.02 ^b^	0.06 ± 0.01 ^a^	0.06 ± 0.02 ^b^	0.16 ± 0.05 ^b^
D-10	0.1 ± 0.01 ^a^	0.13 ± 0.03 ^b^	0.13 ± 0.02 ^b^	0.39 ± 0.31 ^b^	0.10 ± 0.02 ^b^	0.10 ± 0.03 ^b^	0.07 ± 0.02 ^b^	0.24 ± 0.09 ^c^
	*q*CO_2_							
Control	2.18 ± 0.24 ^a^	4.06 ± 1.13 ^a^	4.73 ± 1.87 ^a^	3.53 ± 1.02 ^a^	1.76 ± 0.26 ^a^	1.55 ± 0.48 ^a^	0.79 ± 0.22 ^a^	2.25 ± 0.86 ^a^
D-1	3.21 ± 1.15 ^b^	3.48 ± 0.98 ^a,b^	3.60 ± 0.59 ^a,b^	16.6 ± 9.04 ^b^	2.52 ± 0.45 ^b^	1.56 ± 0.14 ^a^	1.57 ± 0.52 ^b^	3.95 ± 1.23 ^b^
D-10	2.09 ± 0.18 ^a^	3.13 ± 0.73 ^b^	3.24 ± 0.38 ^b^	9.61 ± 3.82 ^b^	2.36 ± 0.41 ^b^	2.50 ± 0.71 ^b^	1.74 ± 0.51 ^b^	5.84 ± 2.12 ^c^
	MB_SIR_/SOC							
Control	0.79 ± 0.13 ^a^	0.55 ± 0.15 ^a^	0.30 ± 0.09 ^a^	0.60 ± 0.17 ^a^	1.11 ± 0.09 ^a^	1.16 ± 0.11 ^a^	1.93 ± 0.345 ^a^	0.62 ± 0.05 ^a^
D-1	1.05 ± 0.14 ^b^	0.56 ± 0.09 ^a^	0.57 ± 0.0 ^b^	0.38 ± 0.08 ^b^	1.57 ± 0.12 ^b^	1.72 ± 0.18 ^b^	1.29 ± 0.16 ^b^	0.34 ± 0.06 ^b^
D-10	0.98 ± 0.14 ^b^	0.70 ± 0.16 ^b^	0.63 ± 0.08 ^c^	0.47 ± 0.11 ^c^	1.52 ± 0.16 ^b^	1.97 ± 0.18 ^c^	1.48 ± 0.13 ^c^	0.32 ± 0.05 ^b^
	*q*CO_2_/SOC							
Control	0.13 ± 0.02 ^a^	0.23 ± 0.06 ^a^	0.27 ± 0.11 ^a^	0.21 ± 0.06 ^a^	0.11 ± 0.02 ^a^	0.10 ± 0.03 ^a^	0.05 ± 0.02 ^a^	0.15 ± 0.06 ^a^
D-1	0.17 ± 0.07 ^b^	0.18 ± 0.05 ^b^	0.19 ± 0.03 ^b^	0.90 ± 0.49 ^b^	0.19 ± 0.04 ^b^	0.10 ± 0.01 ^a^	0.09 ± 0.03 ^b^	0.25 ± 0.07 ^b^
D-10	0.11 ± 0.01 ^c^	0.16 ± 0.04 ^b^	0.16 ± 0.02 ^c^	0.52 ± 0.41 ^b^	0.16 ± 0.03 ^b^	0.17 ± 0.05 ^b^	0.11 ± 0.03 ^b^	0.36 ± 0.12 ^c^

Note: Different letters indicate statistically significant differences (*p* < 0.05) based on Student’s *t*-test or Mann–Whitney U test (Holm-Bonferroni correction).

## Data Availability

The Illumina MiSeq raw reads were deposited in the NCBI Sequence Read Archive (SRA) under the Bioproject PRJNA1107916, BioSample: SAMN49889891-SAMN49889970.

## Supplementary Materials

The following supporting information can be downloaded at: https://www.mdpi.com/article/10.3390/biom15111578/s1, Figure S1. The experimental microcosms; Figure S2. Meteorological conditions for the summer period of 2023 [49,50]; Figure S3. The effect of 2,4-DAPG on the functional activity of soil microbial communities; Figure S4. Spearman’s correlation analysis of the dependence between the distribution of data on the daily average temperature, cumulative precipitation and key microbial parameters. Table S1. Changes in the relative abundance of bacterial ASV at the genus level; Table S2. Changes in the relative abundance of fungal ASV at the genus level; Table S3. Parameters of ecological networks of bacterial communities in laboratory conditions; Table S4. Parameters of ecological networks of bacterial communities in field conditions; Table S5. Parameters of ecological networks of fungal communities in laboratory conditions; Table S6. Parameters of ecological networks of fungal communities in field conditions.

## Abbreviations

The following abbreviations are used in this manuscript:

ASV	Amplicon Sequence Variant
SOC	soil organic carbon
TN	total nitrogen
TC	total organic carbon
BR	basal respiration
SIR	substrate-induced respiration
MB_SIR_	microbial biomass carbon (SIR method)
QR	the coefficient of the microbial respiration
*q*CO_2_	the metabolic coefficient
MB-SIR/SOC	the share of microbial biomass carbon in organic carbon
*q*CO_2_/SOC	the relationship between C-use efficiency and of the available organic carbon in soil

## Appendix A

**Table A1:** Summary of topological properties used to analyze microbial co-occurrence networks.

Topological Properties	Explanation
Node	An individual taxon (ASV) in the network. Node size often reflects its degree of connectivity (number of links)
Link	A connection (co-occurrence or correlation) between two taxa (nodes) in the network
Number of nodes	The total number of taxa (ASVs) included in the network analysis. Reflects taxonomic richness
Number of links	The total number of significant connections between all nodes in the network. Indicates overall network complexity
avgCC	Average Clustering Coefficient. Measures the tendency of nodes to form clusters (how interconnected a node’s neighbors are). High values indicate high local clustering and network stability.
CD	Degree Centralization. Measures how centralized the network is around a few highly connected nodes (hubs). High values indicate network vulnerability.
Power-law (R^2^)	Coefficient of determination for the power-law distribution. Indicates how well the network fits a scale-free model (presence of few highly connected hubs). High R^2^ (close to 1) suggests a stable and robust structure.
max degree	Maximum degree. The highest number of connections held by a single node in the network. Identifies keystone hub taxa.
avgK	Average degree/Mean connectivity. The average number of connections per node in the network. Reflects overall community connectedness.
Density	Network density. The ratio of observed links to the maximum possible number of links. Indicates the overall connectedness of the network.
CB	Betweenness Centrality. Measures how often a node acts as a “bridge” on the shortest path between other nodes. High values indicate a taxon’s key role in maintaining network integrity.
E	Global Efficiency. A measure of how efficiently information or resources are exchanged across the network. A decrease indicates loss of integrity and stability.
Eigenvector centrality	Measures a node’s influence in the network, considering both its own connections and the connections of its neighbors. High values indicate connections to other influential nodes.
Module	A group of nodes that are more densely connected to each other than to nodes in other groups. Reflects ecological guilds or functional groups.
Fragmentation	The breakdown of a single network into isolated or poorly connected modules/components. A sign of strong disturbance.
Trophic plasticity	The ability of an organism or community to switch between different food sources or resource consumption strategies
