# Supplementary material for: From Lab to Field: Context-Dependent Impacts of Pseudomonas-Produced 2,4-Diacetylphloroglucinol on Soil Microbial Ecology
Source: Biomolecules. 2025 Nov 10;15(11):1578. doi: 10.3390/biom15111578 (PMC12650160; doi:10.3390/biom15111578)
Supplement: Supplementary file 1 [file biomolecules-15-01578-s001.zip › biomolecules-3883218-supplementary.pdf]

Supplementary materials

From Lab to Field: Context-Dependent Impacts of Pseudomonas-Produced 2,4-diacetylphloroglucinol on Soil Microbial Ecology

Anastasia V. Teslya [1,2], Artyom A. Stepanov[1], Darya V. Poshvina,[1] Ivan S. Petrushin [3,4] and Alexey S. Vasilchenko[1*]

[1] Laboratory of Antimicrobial Resistance, Institute of Environmental and Agricultural Biology (X-BIO), Tyumen State University, Tyumen, 625003, Russia

[2] Laboratory of Biochemistry and Ecology of Microorganisms, All-Russian Institute of Plant Protection, 196608 Pushkin, Russia

[3] Irkutsk state university, 664033, Russia

[4] Siberian Institute of Plant Physiology and Biochemistry, Siberian Branch of the Russian Academy of Sciences, Irkutsk, 664033, Russia

* Correspondence: avasilchenko@gmail.com (ASV).

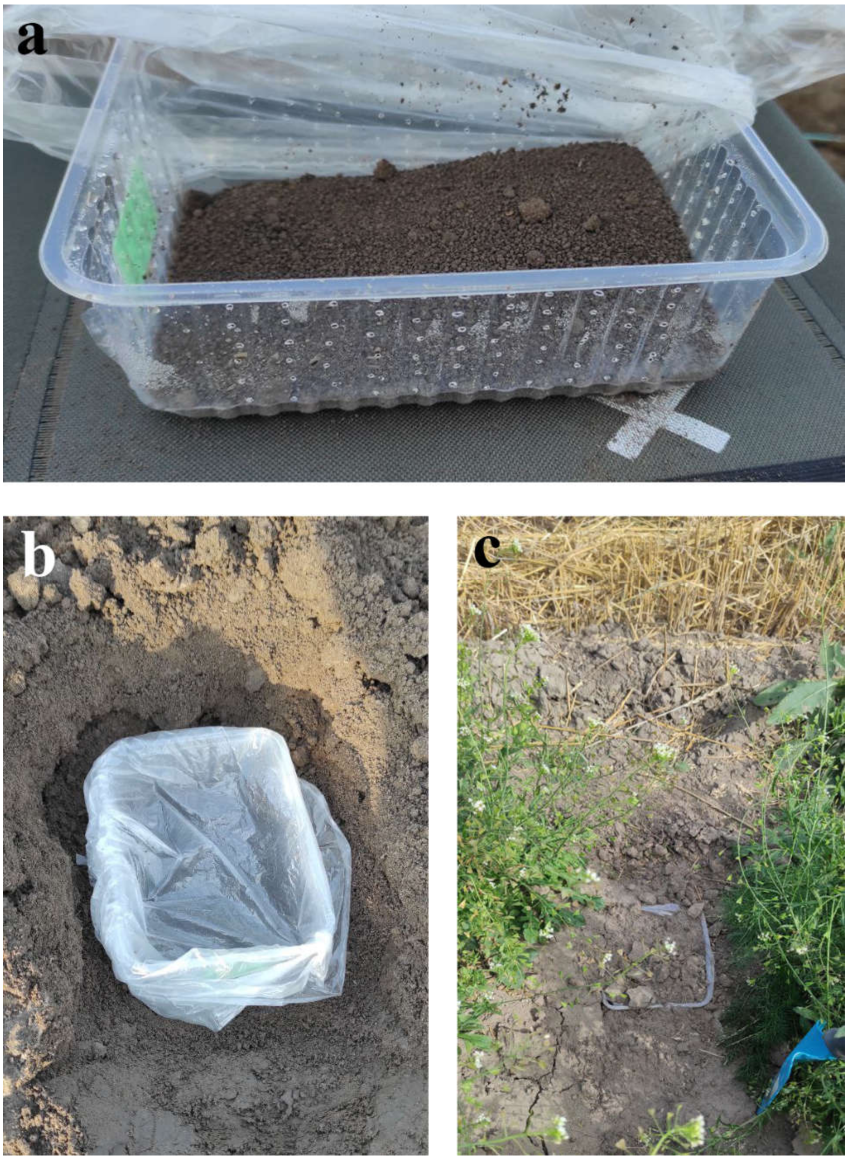

Figure S1. The experimental microcosms. All soil samples were then homogenized by mixing thoroughly for 5 minutes to ensure uniform distribution of 2,4-DAPG solution (a). The water-holding capacity of the soils was adjusted to 60%. Containers with 2,4-DAPG -treated soils were placed at a depth of 0–5 cm in the agrocenosis (where the soil was initially collected) (b, c). Soil samples were collected from each microcosm after 7, 14, 28, and 56 days of incubation.

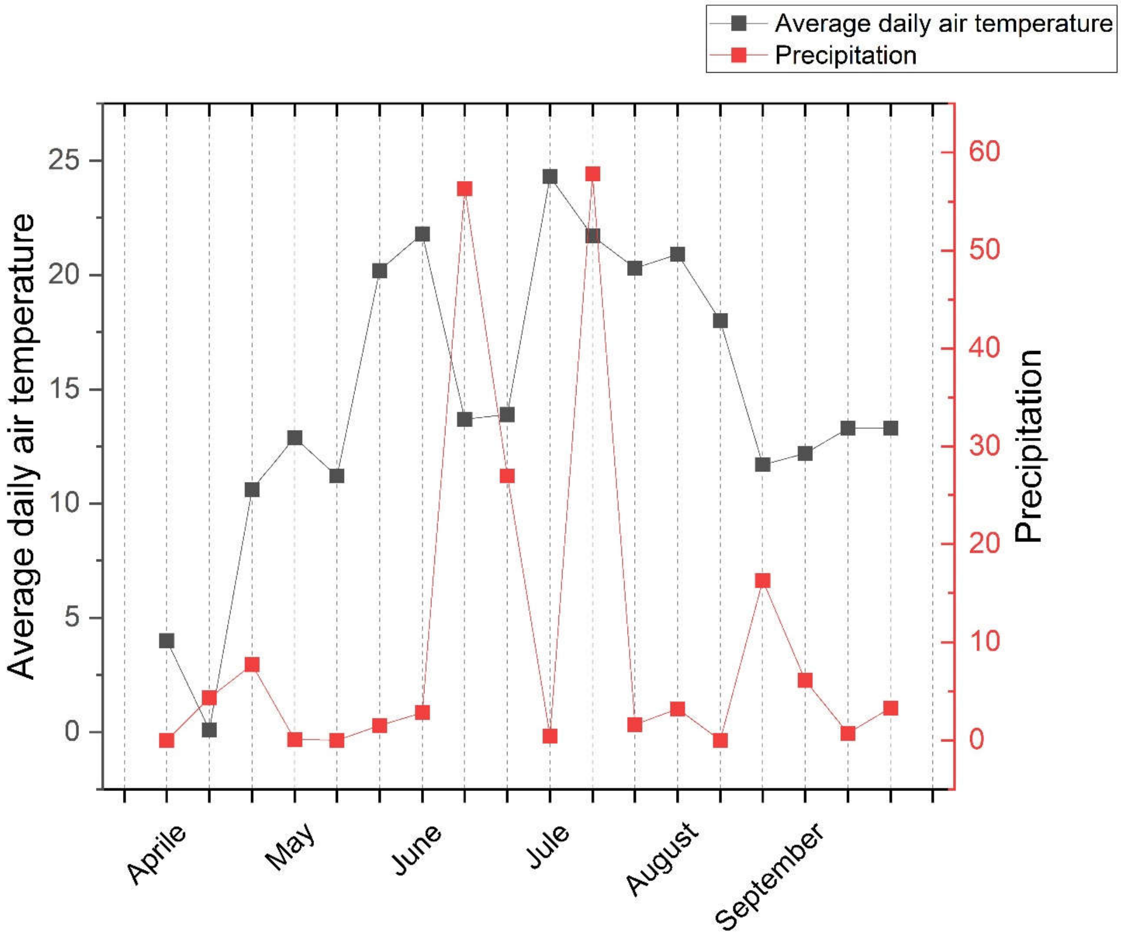

Figure S2. Meteorological conditions for the summer period of 2023. Data are given as average daily air temperature, in degrees Celsius; precipitation in mm. The precipitation deficit was observed during all summer months, except for the second and third ten-day periods of June and the second ten-day period of July. During these periods, precipitation fell by 296%, 123%, and 162%, respectively, compared to the average long-term norm. The Selyaninov hydrothermal coefficient (Selyanoinov 1928; Devyatova, E et al. 2022), which measures drought in terms of heat and moisture, indicates that the second ten-day period of July and third ten-day period of August had favorable moisture conditions.

References

Selyanoinov G.T. About agricultural estimate of climate *Proc. of Agricultural Meteorology*, 1928, 20 165-77

Devyatova E., Kochugova E., Cydenzapov M. Comparison of Selyaninov's Hydrothermal Coefficient (Aridity Criterion) over Buryatia, Russia, in the Summer Period from 1979 to 2019 according to Meteorological Stations and ECMWF ERA5. *Environmental Sciences Proceedings*, 2022, 19*(1)*, 55. https://doi.org/10.3390/ecas2022-12805

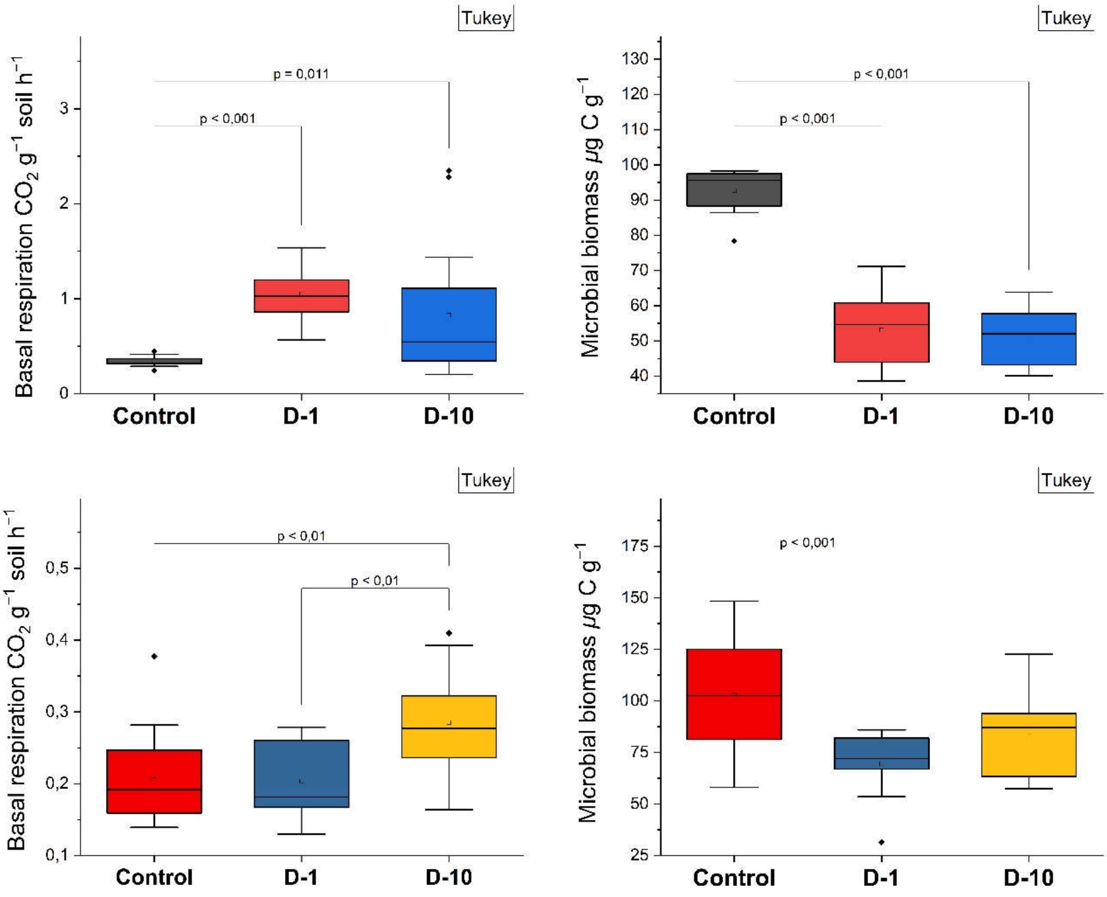

Figure S3. The effect of 2,4-DAPG on the functional activity of soil microbial communities. Dynamics of basal respiration (BR, A, C) and microbial biomass (MB_{SIR}, B, D) under laboratory (A, B) and field conditions (C, D). Data points are means ± standard deviations (n=5). Different letters indicate statistically significant differences between treatments on a 56 day of incubation ($p < 0.05$).

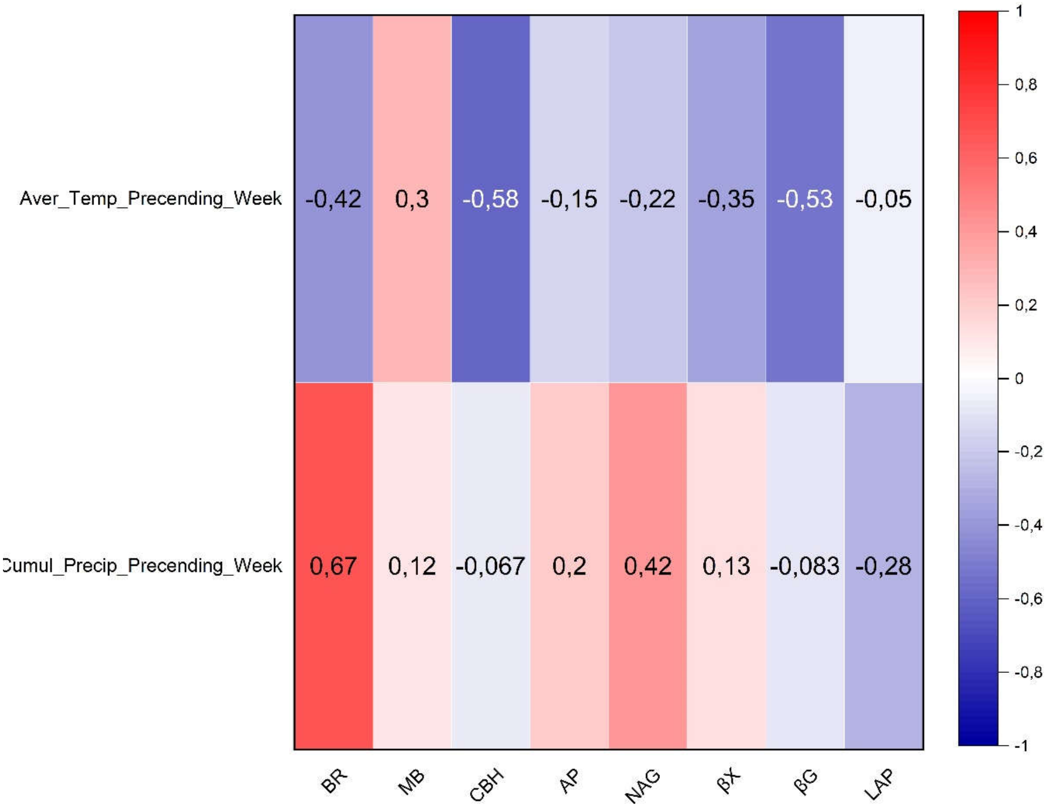

Figure S4. Spearman's correlation analysis of the dependence between the distribution of data on the daily average temperature, cumulative precipitation and key microbial parameters.

Table S1. Changes in the relative abundance of bacterial ASVs in soil microcosms treated with 2,4-DAPG compared to control microcosm are presented. The results are shown as mean log2 fold changes (n=5).

**ASVs**	**Bacterial taxa**	**D-1**	**D-10**
	Laboratory conditions		
ASV_5	*Hyphomicrobium*	10.25	10.956
ASV_107	*Thermoleophilum*	9.47	
ASV_126	*Actinomarinicola*	6.89	
ASV_183	*Gaiella*	6.67	
ASV_182	o_Solirubrobacterales		7.455
ASV_29	*Neobacillus*	5.65	5.677
ASV_63	*Paenibacillus*	2.75	3.102
ASV_122	f_Oxalobacteraceae	1.04	1.630
ASV_18	*Bacillus*	0.59	1.030
ASV_59	*Bacillus*		5.180
ASV_1	*Methylocapsa*		1.928
ASV_46	*Sphingomonas*	0.38	
ASV_69	*Gaiella*	0.36	
ASV_31	*Methylocystis*	-0.19	
ASV_2	*Bradyrhizobium*	-0.39	
ASV_61	o_Rhizobiales	-0.47	

ASV_70	*Nitrospira*	-0.50	
ASV_66	o_Gp3	-0.55	
ASV_149	NA		0.638
ASV_14	Rhizobiales		0.453
ASV_79	NA		0.451
ASV_118	*Skermanella*	-0.57	-2.977
ASV_119	o_Gp3	-4.99	-2.714
ASV_41	o_Gp1	-0.70	
ASV_150	f_Chitinophagaceae		-4.877
ASV_74	*Mesorhizobium*		-5.220
ASV_36	o_Gp1		-5.369
ASV_15	*Pseudarthrobacter*	-3.03	-5.610
ASV_44	f_Bradyrhizobiaceae	-3.21	-5.634
ASV_224	*Rhodoligotrophos*		-6.376
ASV_216	*Nocardioides*		-6.837
ASV_364	*Gaiella*	-4.28	
ASV_139	*Stenotrophobacter*	-4.76	-9.009
ASV_153	o_Gp2	-4.83	
ASV_165	f_Bradyrhizobiaceae	-9.77	-7.291
ASV_101	*Streptomyces*	10.25	-7.427
Field conditions			
ASV_107	c_Spartobacteria	5.36	
ASV_198	c_Spartobacteria	4.46	
ASV_2	*Sphingomonas*		0.53
ASV_19	c_Rhizobiales		0.28
ASV_98	Bradyrhizobiaceae	-5.07	
ASV_60	c_Spartobacteria	-5.48	
ASV_48	Acetobacteraceae	-5.89	
ASV_194	c_Spartobacteria	-6.09	
ASV_127	c_Thermoleophilia	-6.67	
ASV_110	o_Rhodospirillales		-5.07
ASV_48	o_Acetobacteraceae		-7.62
ASV_192	f_Sphingomonadaceae	-4.60	

Table S2: Changes in the relative abundance of fungal ASVs in soil microcosms treated with 2,4-DAPG compared to control microcosm are presented. Results are shown as mean log2 fold change (n=5)

**ASVs**	**Fungal taxa**	**D-1**	**D-10**
Laboratory conditions			
ASV_3	*Plectosphaerella*		-0.34
ASV_6	*Linnemannia*	-1.15	-1.19
ASV_7	*Linnemannia*	-0.57	
ASV_8	*Mortierella*	-0.81	-0.84
ASV_32	o__Pleosporales	0.57	0.46
ASV_38	*Mortierella*	-0.56	
ASV_39	*Neocosmospora*	0.79	
ASV_42	*Albifimbria*	0.49	0.71
ASV_44	*Chaetomium*		0.49

ASV_56	*Metacordyceps*	0.88	
ASV_90	*Penicillium*	4.17	4.215
ASV_58	*Chaetomium*		0.76
ASV_73	*Equiseticola*		0.69
ASV_100	o__Agaricostilbales	0.88	1.29
ASV_110	*Geminibasidium*	5.03	
ASV_147	*Truncatella*		3.39
ASV_124	k__Fungi	1.77	
ASV_151	*Myrmecridium*		3.01
ASV_132	*Saitozyma*	4.34	
ASV_165	f__Nectriaceae		1.62
ASV_143	*Leptosphaeria*	-2.89	
ASV_169	*Colletotrichum*	4.00	3.93
ASV_211	p__Ascomycota	-2.77	
ASV_186	Mortierellales_gen_Incertae_sedis		4.08
Filed conditions			
ASV_28	*Fusicolla*	3.47	
ASV_13	*Chloridium*		0.43
ASV_20	*Saitozyma*		0.40
ASV_6	*Solicoccozyma*		0.20
ASV_4	*Mortierella*		-0.41
ASV_19	*Fusarium*	-0.55	
ASV_136	*Mortierella*	-5.80	
ASV_100	*Leptosphaeria*	-6.83	-8.65
ASV_74	f__Mortierellaceae	-7.22	

Table S3. Parameters of ecological networks of bacterial communities in laboratory conditions

**Network Indexes**	**Control**	**D-1**	**D-10**
Total nodes	61	36	37
Total links	48	35	29
R square of power-law	0.933	0.791	0.976
Average degree (avgK)	1.574	1.944	1.568
Average clustering coefficient (avgCC)	0	0.236	0
Average path distance (GD)	2.125	1.910	1.849
Geodesic efficiency (E)	0.594	0.603	0.661
Harmonic geodesic distance (HD)	1.684	1.657	1.513
Maximal degree	7	13	6
Nodes with max degree	Micrococcales; Mycobacteriales	Nitrosomonadales	Blastocatellales
Centralization of degree (CD)	0.094	0.334	0.130
Maximal betweenness	38	88.333	22.500
Nodes with max betweenness	Gp4	Nitrosomonadales	Blastocatellales
Centralization of betweenness (CB)	0.020	0.147	0.034

Maximal stress centrality	89	102	32
Nodes with max stress centrality	Gp4	Nitrosomonadales	Blastocatellales
Centralization of stress centrality (CS)	0.048	0.169	0.048
Maximal eigenvector centrality	0.378	0.600	0.447
Nodes with max eigenvector centrality	Rhizobiales;Rhizobiales;Myxococcales;Rhizobiales	Nitrosomonadales	Blastocatellales
Centralization of eigenvector centrality (CE)	0.330	0.518	0.382
Density (D)	0.026	0.056	0.044
Reciprocity	1	1	1
Transitivity (Trans)	0	0.265	0
Connectedness (Con)	0.087	0.211	0.110
Efficiency	0.815	0.817	0.739
Hierarchy	0	0	0
Lubness	1	1	1

Table S4. Parameters of ecological networks of bacterial communities in field conditions

**Network Indexes**	**Control**	**D-1**	**D-10**
Total nodes	14	67	55
Total links	9	147	429
R square of power-law	0.992	0.640	0.032
Average degree (avgK)	1.286	4.388	15.600
Average clustering coefficient (avgCC)	0	0.071	0.126
Average path distance (GD)	1.357	3.461	1.820
Geodesic efficiency (E)	0.821	0.356	0.626
Harmonic geodesic distance (HD)	1.217	2.811	1.596
Maximal degree	3	12	28
Nodes with max degree	Streptomycetales	Rhizobiales; Gaiellales	Rhodospirillales
Centralization of degree (CD)	0.154	0.119	0.238
Maximal betweenness	3	652.398	108.167
Nodes with max betweenness	Streptomycetales	Rhizobiales	Nitrospirales
Centralization of betweenness (CB)	0.036	0.270	0.061
Maximal stress centrality	3	6027	1164
Nodes with max stress centrality	Streptomycetales	Frankiales	NA
Centralization of stress centrality (CS)	0.036	2.453	0.447
Maximal eigenvector centrality	0.500	0.283	0.227

Nodes with max eigenvector centrality	*NA;Streptomycetales; NA;NA*	*Geodermatophilales*	*Rhodospirillales*
Centralization of eigenvector centrality (CE)	0.385	0.192	0.101
Density (D)	0.099	0.066	0.289
Reciprocity	1	1	1
Transitivity (Trans)	0	0.065	0.098
Connectedness (Con)	0.154	1	1
Efficiency	0.526	0.948	0.724
Hierarchy	0	0	0
Lubness	1	1	1

Table S5. Parameters of ecological networks of fungal communities in laboratory conditions

**Network Indexes**	**Control**	**D-1**	**D-10**
Total nodes	150	90	82
Total links	3055	130	84
R square of power-law	0.015	0.925	0.865
Average degree (avgK)	40.733	2.889	2.049
Average clustering coefficient (avgCC)	0.477	0.290	0.123
Average path distance (GD)	1.747	2.707	3.012
Geodesic efficiency (E)	0.633	0.498	0.472
Harmonic geodesic distance (HD)	1.579	2.009	2.119
Maximal degree	85	20	8
Nodes with max degree	*Neocosmospora*	*Exserohilum*	*Naganishia*
Centralization of degree (CD)	0.301	0.197	0.075
Maximal betweenness	224.345	179	169.083
Nodes with max betweenness	*Solicoccozyma*	*Oidiodendron*	*Naganishia*
Centralization of betweenness (CB)	0.015	0.043	0.050
Maximal stress centrality	3595	196	390
Nodes with max stress centrality	*Neocosmospora*	*Rhynchogastrema*	*Naganishia*
Centralization of stress centrality (CS)	0.245	0.046	0.114

Maximal eigenvector centrality	0.160	0.459	0.531
Nodes with max eigenvector centrality	*Neocosmospora*	*Exserohilum*	*Naganishia*
Centralization of eigenvector centrality (CE)	0.088	0.418	0.490
Density (D)	0.273	0.032	0.025
Reciprocity	1	1	1
Transitivity (Trans)	0.477	0.298	0.152
Connectedness (Con)	1	0.167	0.126
Efficiency	0.731	0.856	0.870
Hierarchy	0	0	0
Lubness	1	1	1

Table S6. Parameters of ecological networks of fungal communities in field conditions

**Network Indexes**	**Control**	**D-1**	**D-10**
Total nodes	80	61	78
Total links	130	93	137
R square of power-law	0.658	0.833	0.797
Average degree (avgK)	3.250	3.049	3.513
Average clustering coefficient (avgCC)	0.196	0.224	0.240
Average path distance (GD)	6.443	3.097	4.138
Geodesic efficiency (E)	0.244	0.394	0.327
Harmonic geodesic distance (HD)	4.101	2.536	3.055
Maximal degree	9	20	14
Nodes with max degree	*Solicoccozyma*	*Furcasterigmium*	*Albifimbria; Talaromyces*
Centralization of degree (CD)	0.075	0.292	0.140
Maximal betweenness	1099.753	679.851	696.976
Nodes with max betweenness	*Plectosphaerella*	*Furcasterigmium*	*Pleotrichocladium*
Centralization of betweenness (CB)	0.298	0.367	0.216
Maximal stress centrality	6483	1783	2578
Nodes with max stress centrality	*Leptodontidium*	*Furcasterigmium*	*Pleotrichocladium*
Centralization of stress centrality (CS)	1.831	0.953	0.787
Maximal eigenvector centrality	0.329	0.517	0.391
Nodes with max eigenvector centrality	*Solicoccozyma*	*Furcasterigmium*	*Pleotrichocladium*

Centralization of eigenvector centrality (CE)	0.260	0.438	0.338
Density (D)	0.041	0.051	0.046
Reciprocity	1	1	1
Transitivity (Trans)	0.209	0.155	0.258
Connectedness (Con)	0.903	0.648	0.602
Efficiency	0.967	0.943	0.943
Hierarchy	0	0	0
Lubness	1	1	1
